# Sequential autoclave–hydrothermal, enzymatic conversion of cauliflower waste Xylan into high-purity bioactive Xylooligosaccharides: Purification, prebiotic activity and toxicity analysis

**DOI:** 10.1016/j.fochx.2026.104428

**Published:** 2026-09-10

**Authors:** Piyush Verma, Amit Kumar, Ravinder Kaushik, Pooja Dhiman, Chuanling Si, Chin Wei Lai

**Affiliations:** a School of Health Sciences and Technology, UPES, Dehradun, Uttarakhand 248007, India; b Nanotechnology & Catalysis Research Centre (NANOCAT), Institute for Advanced Studies (IAS), University of Malaya (UM), 50603 Kuala Lumpur, Malaysia; c International Research Centre of Nanotechnology for Himalayan Sustainability (IRCNHS), Shoolini University, Solan 173229, India; d State Key Laboratory of Bio-Based Fiber Materials, Tianjin Key Laboratory of Pulp and Paper, Tianjin University of Science and Technology, Tianjin 300457, People's Republic of China; e Faculty of Engineering and Technology, Sunway University, Jalan Universiti, Bandar Sunway, Selangor, Petaling Jaya 47500, Malaysia

**Keywords:** Greener methods, Food waste, Enzymatic hydrolysis, Membrane purification, Prebiotic activity, Xylooligosaccharides, Toxicity

## Abstract

The sustainable valorization of food wastes into health-promoting products as prebiotic xylo-oligosaccharides (XOS) is surely a circular bioeconomy initiative. This study reports autoclave-hydrothermal pretreatment (AC-PT) for superior 71.7% xylan recovery from cauliflower waste. The optimized enzymatic hydrolysis of xylan produced 64.27% XOS, primarily X2 (38.4%), X3 (24.9%), X4 (34.3%) and X6 (2.4%). The sequential ultra- and nano-purification primarily purified the XOS fractions, X2 (55.8%), X3 (35.7%), X4 (8.3%) and X6 (0.2%). The purified XOS showed a good prebiotic effect, promoting the growth of *Lactobacillus* strains with a *> 1* prebiotic index, prebiotic score and good antimicrobial activity. In-vitro XOS fermentation generated lactic acid and SCFAs. The cytotoxicity of XOS exhibited no plant toxicity or cytotoxicity on NCM 460 human cell lines. This work confirms the practical applicability of sequential autoclave hydrothermal pretreatment-enzymatic hydrolysis-membrane purification from cauliflower waste as high-quality prebiotics.

## Highlights

• Autoclave assisted high yield xylan extraction from underutilized cauliflower waste.
• Pretreatment and enzymatic hydrolysed by response surface methodology for best yield.
• High XOS yield of 64.27% was achieved with low enzyme loading and shorter time.
• Sequential ultra and nano-membrane technology-High purity X2-X3 fractions of XOS.
• Excellent prebiotic potential for 5 probiotic cultures- improved gut barrier function.

## Graphical abstract

**Figure:**
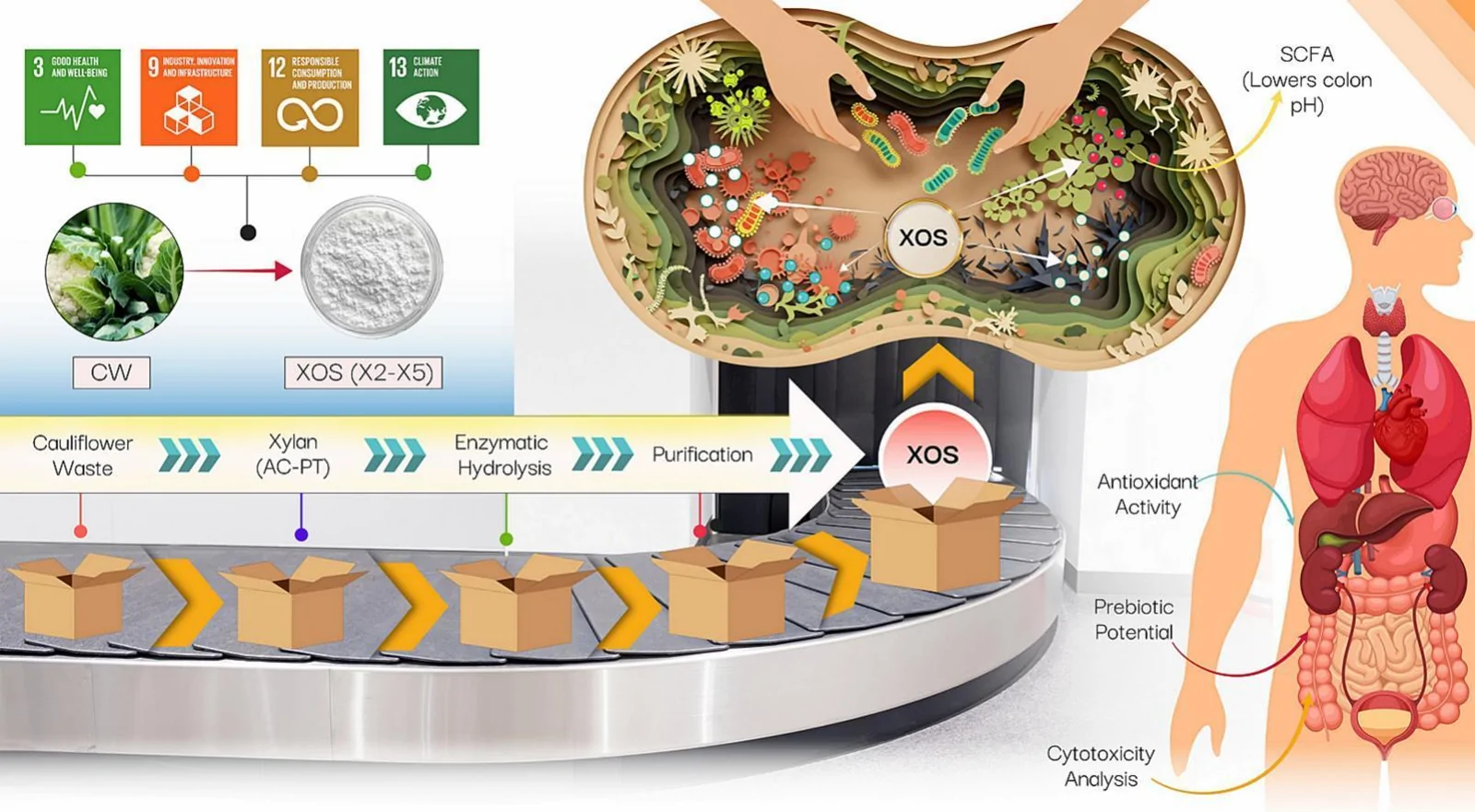

## 1 Introduction

The global population growth has led to a large increase in waste biomass generation, which has increased the consumption of food and other necessities (Ezeorba et al., 2024). The FAO reports that about 1.3 billion tons of food, or greater than one-third of the food generated globally, is lost every year (Bakare et al., 2025). Annually, the world produces more than 5 billion tons of agricultural residue, with Asia (47%), trailed by America (29%), and others, such as Europe (16%) and Africa (7%) (Shinde et al., 2022). Food loss during production and post-harvest is 24% (Chauhan et al., 2021). Inefficient farming and post-storage are the main causes. Transportation generates 17% of waste, and retailers squander 20% of food due to cosmetic standards and overstocking. However, the consumption stage causes the biggest loss (35%) (Onyeaka et al., 2025). This waste's improper disposal causes environmental and economic problems, such as greenhouse gas emissions, water and soil contamination, health hazards and resource loss (Rani & Gwal, 2025). Due to climate change, limited resources, biodiversity loss, and the need for sustainable economic systems, agro-food waste is becoming a valuable resource in the circular bioeconomy (Pandey et al., 2025).

Cruciferous vegetables, widely consumed, exhibit substantial loss across their supply chain, mainly cabbage, cauliflower, and broccoli (Shinali et al., 2024). Cauliflowers and cabbages generate between 30% and 50% of the overall waste (Mago et al., 2022). These wastes are underutilized due to insufficient processing techniques, especially for recovering health-promoting bioactivities, such as dietary fibres and phytochemicals (Verma, Kumar, et al., 2025). Dietary fiber is widely utilized in food and medicine and offers great promise as a food additive or functional food ingredient that addresses the technological demands of value-enhanced wellness products (Maqbool et al., 2025). The circular economy provides a sustainable substitute by targeting zero pollution and waste and turning cauliflower waste into useful products such as Xylooligosaccharides (XOS). The xylo-oligosaccharides refer to non-digestible ones, consisting of sugar oligomers comprising xylose monomers (2–6) connected by β (1 →  4)-xylosidic bonds. The hydrolysis of xylan produces short-chain XOS, specifically xylobiose, xylotriose, and xylotetraose (Chaiyates et al., 2025). They've been shown to have antihypertensive, antidiabetic, and prebiotic properties, as well as to inhibit human colon cancer cells (Ávila et al., 2026). Xylan acts as the principal raw material for XOS via pretreatment and enzymatic hydrolysis. Human and animal studies have revealed that XOS increases *Lactobacillus* and *Bifidobacterium* populations, improves faecal moisture, and lowers intestinal pH, emphasizing its benefits for gut health and increasing its application in functional foods and nutraceuticals (Sabanci et al., 2025). The food industry is one of the main reported applications for XOS, followed by other health and medical applications (Kumar et al., 2024). The modest sweetness (40% as sweet as sucrose) of XOS makes them appropriate for low-calorie items. They are currently classified as emerging prebiotics in several nations, and the FDA has notified them as generally recognized as safe (GRAS) for use in food products in the USA (Verma, Kaushik, & Sirohi, 2025). There is significant promise for lignocellulosic biomass as a substrate source for XOS production. Cauliflower waste (CW) is a notable agricultural waste and can serve as a sustainable resource for xylooligomers production.

The literature reveals a lack of extraction of hemicellulose and further Xylooligosaccharides production from cauliflower waste to develop prebiotics. Secondly, only a few reports have evaluated the impact of steam assistance on xylose recovery, although major research to date has concentrated on the chemical pretreatment of CW. Thus, we hypothesize that autoclave hydrothermal treatment could extract xylose to a higher extent and further sequential enzymatic treatment and ultra/nano purification would yield ultrapure XOS with high prebiotic activity. The goal of this research work is to utilise autoclave-hydrothermal pretreatment (AC-PT) to extract hemicellulose, followed by enzymatic hydrolysis to recover XOS using xylanase. Under optimal conditions, the green process was optimized utilizing the response surface methodology (RSM) to enhance the XOS yield, followed by its purification, antimicrobial/antioxidant, and prebiotic potential assessment, in-vitro digestibility and plant/human cell line toxicity, which will undoubtedly provide current research with a new perspective. This initiative will support a circular bioeconomy by helping to create sustainable and green bio-extraction techniques for bio-valuation of cauliflower waste.

## 2 Materials and methods

### 2.1 Chemicals

All analytical and microbiological-grade reagents and solvents were used in this work. Dinitro salicylic acid (98%), phenol (99%), ethanol (99.9%), methanol (99.8%), acetone (99.5%), xylose and glucose were obtained from SRL, Mumbai, India. The source of recombinant xylanase expressed in *Aspergillus oryzae* (2500 U/g) was Sigma-Aldrich Corporation (Missouri, USA). The strain was procured from the MTCC & Gene Bank in Chandigarh, India, and assigned accession numbers: the LAB cultures (*Lactobacillus plantarum* (MTCC 9511), *Lactobacillus fermentum* (MTCC 903), *Lactobacillus brevis* (MTCC 4460), *Lactobacillus rhamnosus* (MTCC 1408), *Lactobacillus casei* (MTCC 12468), *Staphylococcus aureus* (MTCC 3160), *Bacillus cereus* (MTCC 8527), *Salmonella* spp. (MTCC 3217), and *Escherichia coli* (MTCC 1302) were produced in the MRS medium (MRS, Hi-media Lab Pvt. Ltd., India). Beef extract, polysorbate 80 and Protease peptone (Hi-media Lab Pvt. Ltd., India), sodium acetate (Qualigens), dextrose (Merck Life Science, India), manganese sulfate, triammonium citrate, magnesium sulfate and activated charcoal (Loba Chemie Pvt. Ltd.), yeast extract (Sigma-Aldrich), and dipotassium phosphate (RANKEM) were obtained and utilized for comparative growth analysis. Mueller–Hinton agar and Gentamicin (1 mg/mL) (Sigma-Aldrich, St. Louis, MO, USA) for the antimicrobial activity test. Normal human colon epithelial cells (NCM460) were obtained from a certified cell repository, and the cells were cultured in the standard DMEM supplemented with 10% FBS and 1% penicillin and streptomycin, and maintained at a temperature of 37 °C in a humidity-controlled incubator containing 5% atmospheric carbon dioxide. The cells underwent subculture at 70–80% confluency using trypsin–EDTA.

### 2.2 Sample preparation

**Fig. 1:**
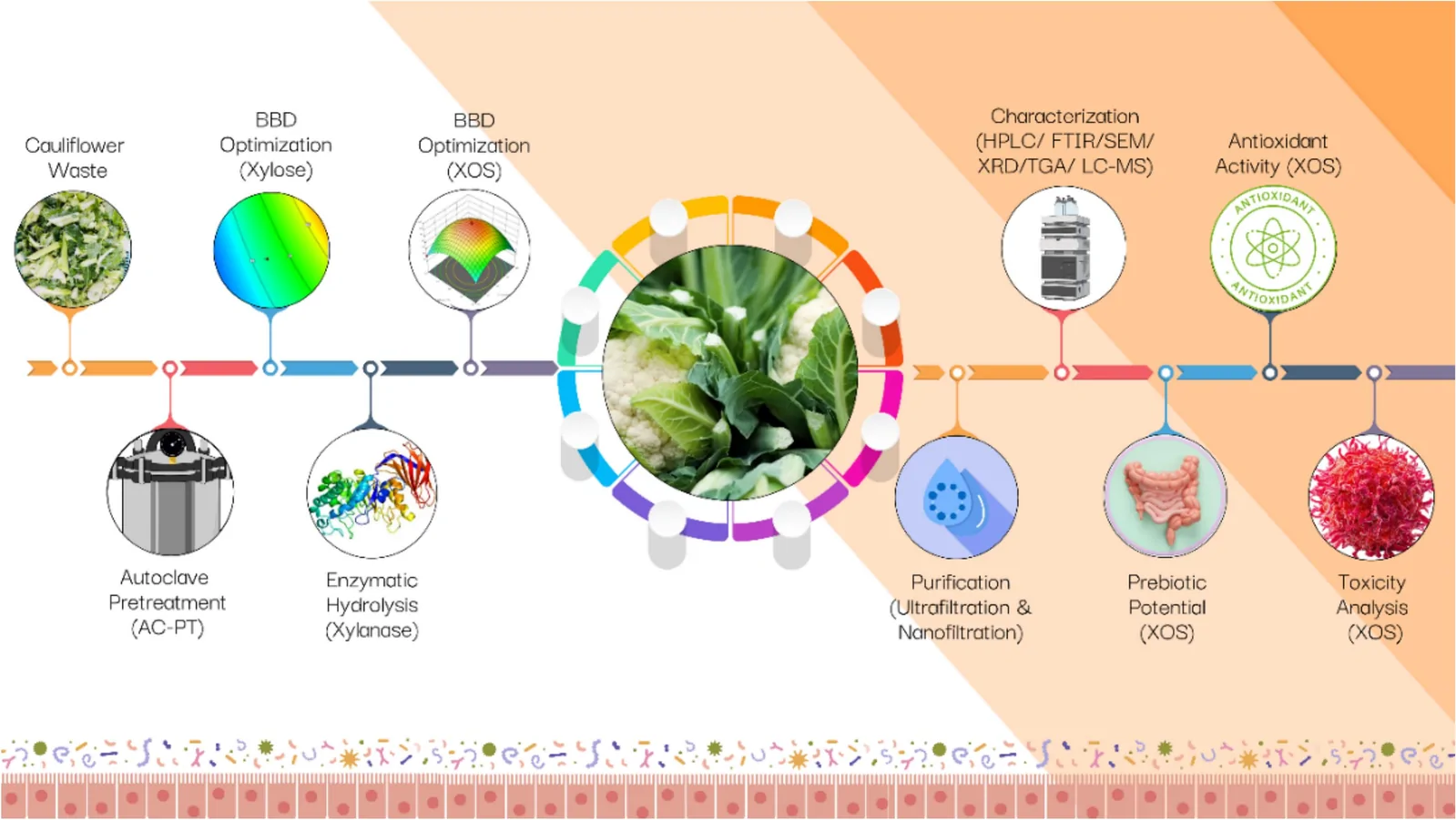
Workflow illustrating the conversion of CW into XOS and the associated pretreatment-hydrolysis optimization, characterization, purification and biological evaluation

The cauliflower (*Brassica oleracea* var. *botrytis*) waste was sourced from the local market in Dehradun, India. The waste was carefully washed with water three times to remove foreign particles, dirt and dust. Subsequently, the CW was oven-dried at 60 °C in a tray dryer for 12 h (DELLMARC, Kerala, India). The dry feedstock was next ground into a powdered material using a grinder at a moderate speed and sieved using a British conventional sieve in order to get particles smaller than 40 μm. Subsequently, it was preserved at ambient temperature (25 °C) in an airtight zip-lock bag until utilized and named as CW. Fig. 1 illustrates the preparation of CW, BBD-based optimization of pretreatment and enzymatic hydrolysis, membrane purification (ultrafiltration and nanofiltration details are provided in the materials section) and prebiotic study thereof.

### 2.3 Extraction procedure: Extract free CW and autoclave-hydrothermal pretreatment (AC-PT)

1 g of CW powder was kept in a 20 mL acetone: ethanol mixture, i.e., 1:2 (*v*/v), at 30 °C for 24 h to produce extractive-free CW. After subsequent filtration, the CW residue was rinsed using hot water. The sample was cleansed multiple times with distilled water (DW) and further underwent additional drying at 45 °C in a hot air oven (Model BPI-9, Ambassador, New Delhi, India) for 24 h to produce extractive-free CW powder, and then processed with AC-PT (Klangpetch et al., 2022). The AC-PT was carried out by mixing the extractive-free CW with distilled water. The AC-PT was carried out for 10, 20, and 30 min for each parameter at temperatures of 100, 150, and 200 °C with S: L ratios (1:20, 1:40 and 1:60). The experimental mixture was subsequently allowed to cool to 25 °C. Following that, vacuum filtration was used to separate the liquid supernatant from the particles. 95% ethanol was used to wash the prepared CW residue. After that, it was cleaned with DW and allowed to dry in an oven overnight at 45 °C (Model BPI-9, Ambassador, New Delhi, India). The surface characteristics and thermal stability were then examined using the residue. Centrifugation using a refrigerated centrifuge (CPR-24 Plus) (Remi Elektrotechnik Ltd., Mumbai, Maharashtra, India) was used to further separate the liquid fractions for 20 min at 4 °C and 9000 rpm, and the produced supernatant was subsequently stored at −20 °C (TRUFROST UF-371 Premia, India) until further use. The BBD-based statistical experimental design for autoclave hydrothermal pretreatment details is provided in the supplementary file **(Table S1).**

The dinitrosalicylic acid method (DNS) was employed to quantify reducing sugar (xylose) in CW-AC-PT for optimization, using xylose as the standard (Miller, 1959). The total sugars were analyzed by the phenol‑sulfuric technique, with glucose serving as the standard (DuBois et al., 1956). The xylose in the hemicellulose fraction from the autoclave pretreatment was determined by HPLC (Agilent 1260, Santa Clara, USA) using a Metrohm Metrosep Carb 2 column (150 mm × 4.0 mm) equipped with an RI detector. Milli-Q water was used as the mobile phase (0.6 mL/min flow rate) for sugar determination, and a column temperature of 80 °C.

### 2.4 Enzymatic hydrolysis for XOS extraction

Enzymatic hydrolysis (EH) of the AC-PT xylan-rich hemicellulose fraction was performed using the xylanase enzyme (2500 U/g). The enzymatic hydrolysis was performed at 40, 50 and 60 °C with continuous shaking on an orbital shaker (REMI, CIS-24 Plus, Mumbai, India) (150 to 200 rpm) for 12, 30 and 48 h at three different enzyme doses of 15, 25 and 35 U and at a constant pH (5). The effects of enzyme concentration, incubation temperature and duration were determined by employing the XOS (%) produced as the efficacy indicator. The BBD-based statistical experimental design information is provided in the supplementary file **(Table S1).**

A pH adjustment to 6.5 was performed on the extract produced after EH, which was then subjected to ultrafiltration and nanofiltration sequentially. Initially, large molecular weight (>10 kDa) compounds were eliminated using ultrafiltration (UF) with a 10 kDa membrane attached (Amicon Ultra-4) based on regenerated cellulose. The initial pH of the hydrolysate obtained was adjusted to 6.5. The ultrafiltration tubes were first dipped in deionized water for water passage and then kept at 4 °C for 10 min. Further, 5 mL of hydrolysate was transferred to the UF tube. At this stage, the retentate was discarded, and the permeate fraction was collected. Further, the UF permeate underwent a pH change (3.8) and was submitted to the nanofiltration (NF) membrane (NFX) (Synder) to eliminate monosaccharides and other lower MW molecules with an MW of 100–200 Da. After NF, the retentate was collected, and the permeate was discarded. The temperature was kept at 20 °C, and the pressure was kept at 20 bar in each case (Centeno et al., 2023). The temperature was 20 °C, and the pressure was 20 bar for UF and 30 bar for NF.

XOS content after enzymatic hydrolysis and membrane purification was measured by HPLC (Waters 515 HPLC System, Milford,909 Massachusetts, USA). The column used was a Waters Spherisorb NH_2_ column (4.6 mm × 250 mm) equipped with a Waters 2414 RI detector. For XOS estimation, a mobile phase of Acetonitrile: HPLC Water (63:37) was employed at a flow rate of 0.5 mL/min, and the column temperature was set at 30 °C. XOS before and after purification was also characterized using MALDI–TOF mass spectrometry (detailed method in supplementary data S2.2).

### 2.5 Prebiotics potential assessment by in-vitro fermentation assay

Protease peptone (1 g), polysorbate 80 (0.1 g), yeast extract (0.5 g), 1 g beef extract, CH_3_COONa (0.5 g), K_2_HPO_4_ (0.2 g), triammonium citrate (0.2 g), MgSO_4_ (0.01 g) and MnSO_4_ (0.005 g) were mixed to form the modified MRS base, excluding any carbon source. The XOS at different concentrations (1 and 2 mg/mL) was added to the MRS base. The growth stimulation was compared to glucose as a control. The growth-promoting impact of the produced XOS was examined using the standard plate count according to the specified methodology (Sonkar et al., 2023) with minor modifications. The three media used for the XOS fermentation experiment were XOS mixture as a C-source (test), glucose as a C-source (+ve), and no C-source (−ve). The LAB cultures (*L. plantarum* (MTCC 9511), *L. rhamnosus* (MTCC 1408), *L. brevis* (MTCC 4460), *L. casei* (MTCC 12468), *L. fermentum* (MTCC 903), and *E. coli* (MTCC 723)) were cultured separately in the MRS medium and anaerobically maintained for 24 h at 37 °C. Using a colony counter, the number of colonies was determined following incubation, and the growth was computed as log_10_CFU/mL.

### 2.6 Lactic acid levels measurement and SCFA analysis

The lactic acid level generated during in-vitro fermentation, i.e. total lactic acid titrated (TLAT), was tested by the standard titration method using 0.1 N NaOH. In a typical process, 5 mL of the broth was used for measurement. Further short-chain fatty acid (SCFA) analysis was conducted by GC-FID. The SCFAs from fermentation broth after freezing of microbes were converted into FAMEs. 1000 μL of sample from broth was added to 5 mL Teflon-lined glass vials, and internal standard isobutyric acid was dissolved in hexane. After transferring to the rotary evaporator at 35 °C, anhydrous methanolic HCl was added and incubated for 2 h. The contents were cooled and washed with 1 M NaOH and 2% NaHCO_3_ and recovered in hexane to form FAMEs. The FAMEs were then analyzed by gas chromatography on an Agilent 7890B, Canada, Inc., instrument equipped with an autoinjector and flame ionisation detector (FID).

### 2.7 Prebiotic index/score calculation

The prebiotic index (PI) was calculated as per the standard method (Palframan et al., 2003). PI is termed the ratio of probiotic growth in prebiotic (like the XOS in this case) to probiotic growth in control carbohydrate (glucose in this case). The prebiotic index was determined as per Eq. 1:

(1) $$\mathrm{PI}=\frac{\mathrm{CFU}\;\mathrm{of probiotics in prebiotic}\;\mathrm{XOS}}{\mathrm{CFU}\;\mathrm{ofprobiotics in control carbohydrate}}$$

Prebiotic activity score (PS) was analyzed by eq. 2, as reported by (Huebner et al., 2007; Palframan et al., 2003):

(2) $$\mathrm{PS}=\frac{{\left(\mathrm{Log}P_{24}-\mathrm{Log}P_{o}\right)}_{\mathrm{prebiotic}}}{{\left(\mathrm{Log}P_{24}-\mathrm{Log}P_{o}\right)}_{\mathrm{control}}}-\frac{{\left(\mathrm{Log}E_{24}-\mathrm{Log}E_{o}\right)}_{\mathrm{prebiotic}}}{{\left(\mathrm{Log}E_{24}-\mathrm{Log}E_{o}\right)}_{\mathrm{control}}}$$

Log P24 denotes the logarithm of growth (CFU/mL) of probiotic bacteria at 24 h, and Log P_0_ indicates the growth at 0 h in both prebiotic and control conditions. The logarithm of *E. coli* growth (CFU/mL) at 24 h (E24) and 0 h (E0) under prebiotic and control environments is referred to as log E.

### 2.8 Prebiotic digestibility: Simulation

The resistance of purified XOS to enzymes involved in the digestive process was further tested by in-vitro digestibility tests through the simulated oral phase, the gastric phase, the intestinal phase, and the Brush Border Membrane Vesicles (BBMV) phase (Detailed process in supplementary data S 2.3).

### 2.9 Toxicity analysis, antimicrobial and antioxidant potential

The toxicity of XOS was studied by standard plant toxicity and human cell line cytotoxicity (cell viability). The procedure details are in the supplementary data (S2.6 & S2.7). The details of antimicrobial and antioxidant activity experiments are also provided in the supplementary data (S2.8 & S2.9).

### 2.10 statistical analysis

Statistical evaluation of the design matrix was performed using multiple non-linear regression, further subjected to ANOVA using RSM, via Design Expert-13 software (v13.05.0), developed by State-Ease, Minnesota, USA, in accordance with the F-test. The model underwent evaluation for appropriateness using the lack-of-fit test. Tukey's post hoc test was employed to analyse standard deviation (±SD) as well as significant differences (*p* < 0.05).

## 3 Results & discussion

### 3.1 Xylose extraction from CW by autoclave-hydrothermal treatment

#### 3.1.1 Effect of BBD-based AC-PT conditions on xylose yield

The biomass composition and nutritional profile of cauliflower waste used for extraction of xylose were also determined. The biomass consisted of 26.2 ± 0.21% cellulose, 17.0 ± 0.14% hemicellulose and 2.56 ± 0.16% lignin. Additionally, the nutritional composition of CW shows 16 ± 0.18% protein, 38.02 ± 0.16% carbohydrate and 31.55 ± 0.31% fiber. The waste and discarded parts of cauliflower were used to prepare the CW powder and further used for the extraction process. The obtained xylose and total sugar concentrations from CW are significantly influenced by operational parameters, i.e., S: L ratio, time and temperature, for autoclave-assisted pre-treatment. The RSM technique was utilized to optimize the process and operational conditions, and 17 experimental runs were conducted (BBD method). Each factor's levels for BBD-based RSM, along with the expected and actual results (in terms of xylose/total sugar yield) for each experimental run, are presented in the supplementary file **(Table S2).** The yield of AC-PT extraction-assisted xylose ranged from 27.07 to 99.13 mg/g. The total sugar extracted ranged from 32.21 to 126.13 mg/g over a temperature range of 100–200 °C and an extraction duration of 10–30 min. The highest xylose yield and total sugar yield were 99.13 mg/g and 126.13 mg/g, respectively (200 °C, 20 min, and 1: 60) **(Table S2)**. The interactions and contour plots for total sugar and reducing sugar (xylose) in CW after AC-PT are given in supplementary data **(Fig. S1 (a-b)).** The ANOVA analysis is provided in the supplementary data and **Table S3**.

#### 3.1.2 Xylose extraction analysis by HPLC

HPLC analysis was performed using the liquid extract (after AC-PT) collected under the specified conditions (as optimized under RSM conditions and subsequent DNS technique), i.e. for CW-AC-PT, and an S: L ratio of 1: 60, a temperature of 200 °C and a duration of 20 min were used. The HPLC chromatogram is shown in the supplementary file **(Fig. S2 (a)).** HPLC investigation into pretreatment CW revealed that the xylose concentration was 9.24 mg/mL for CW-AC-PT. The results showed that 71.7% of xylose could be extracted from biomass by AC-PT. 1 g of cauliflower waste with 17.0% xylan was equivalent to 193.18 mg of theoretical xylose, whereas 138.60 mg of xylose was extracted experimentally from 15 mL of hydrolysate, yielding a 71.7% xylose extraction efficiency. Furthermore, the high yield is achieved under ambient AC-PT conditions. Autoclave pretreatment offers a superior method for the extraction of xylose due to its significantly higher xylose yield. In addition to its efficiency, AC-PT offers a green and sustainable approach, as it employs water as a solvent, avoids harsh chemical use and operates under controlled conditions. CW-AC-PT combines both high extraction efficiency and environmental sustainability, thereby paving the way for its potential application in food-grade xylose and value-added product development. The xylose extraction efficiency from CW is quite high, which is beneficial and important owing to the very low content of hemicellulose in it. As a vegetable, cauliflower has a waste part which is quite underutilized and poorly valorised; the extraction of xylose and further XOS is highly relevant and promising.

#### 3.1.3 Structural analysis

**Fig. 2:**
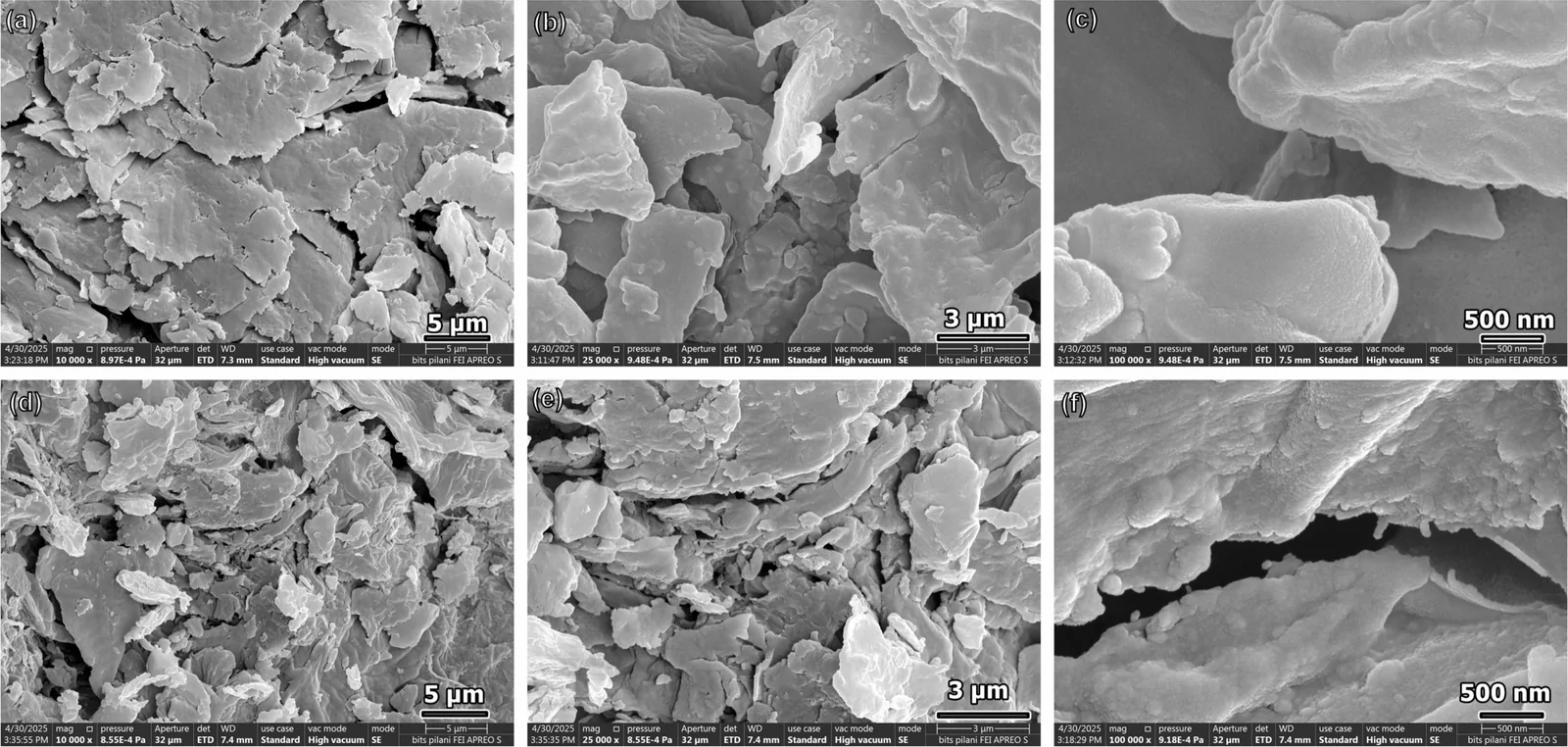
SEM images of (a-c) CW (Untreated), (d-f) AC-PT.

Structural analysis was performed on the CW powder after the extraction of xylose by AC-PT to confirm the extraction. The SEM images for untreated biomass, i.e. CW and treated biomass, i.e. AC-PT, are shown in Fig. 2**(a-f).** The micrograph for raw CW shows a smooth and tight surface with dense layering. In the raw biomass, cellulose chains are well-oriented and amalgamated together by hemicellulose, in a complex arrangement forming a relatively smooth texture. The AC-PT leads to erosion of the CW surface owing to damage to the secondary cell wall layer. The surface thus appears rougher and relatively loose due to the extraction of xylose/hemicellulose.

**Fig. 3:**
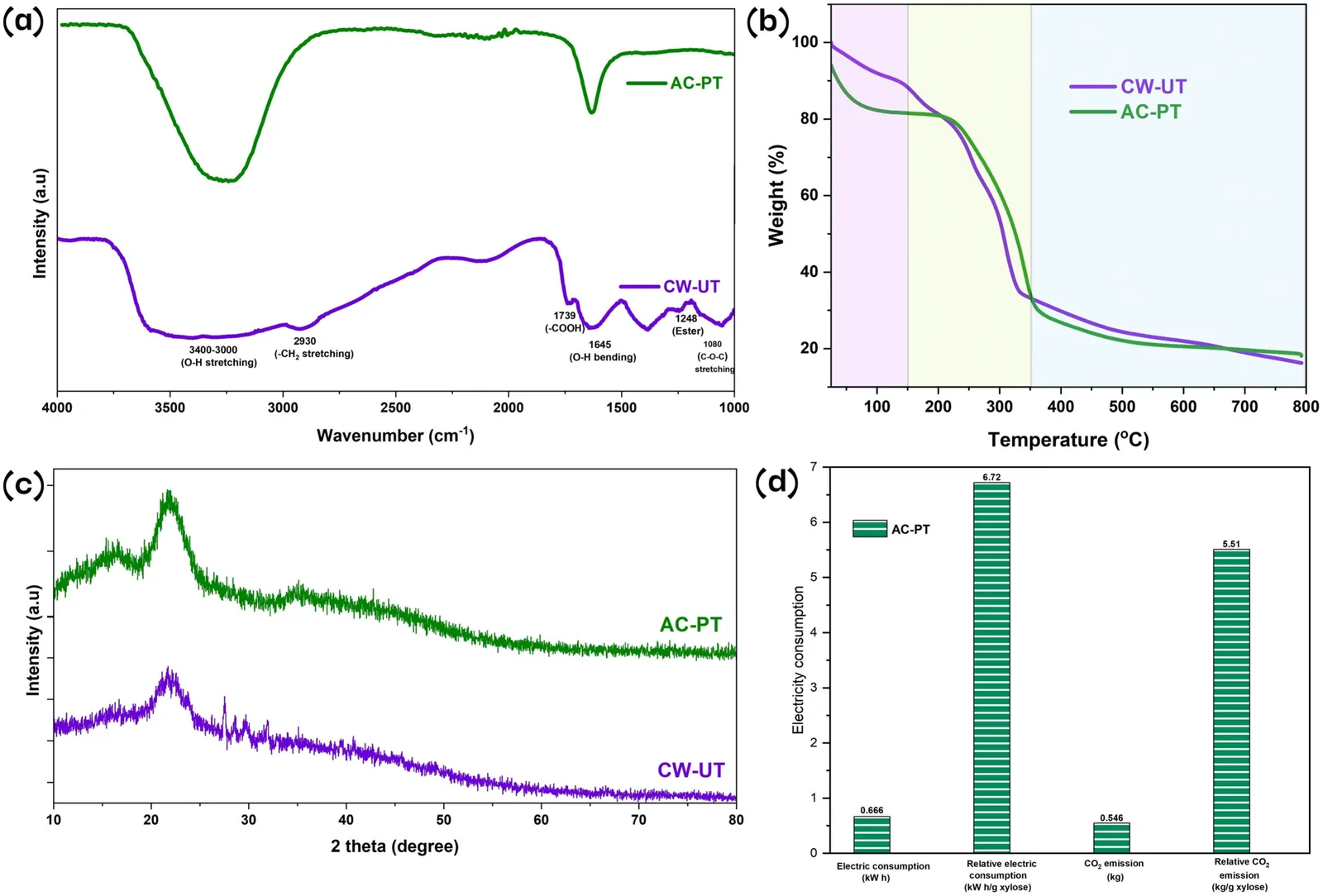
(a) FTIR spectra, (b) TGA thermograms, (c) XRD diffractograms, (d) Electric energy consumption for AC-PT and relative CO_2_ emissions.

Further, FTIR spectra of untreated cauliflower waste, i.e. CW-UT and the pretreated sample, i.e. AC-PT, were obtained and presented in Fig. 3(a)**.** For both untreated and pretreated samples, the broad bands between 3400 and 3000 cm^−1^ were observed for O—H stretching vibrations (Sadeghi-Shapourabadi et al., 2023). In addition, for CW, typical -CH_2,_ i.e. methylene symmetric stretching peaks, were noted at nearly 2930 cm^−1^ of the hemicellulose fraction and cellulose portion in raw biomass. The observed characteristic peak at 1645 cm^−1^ refers to water bending (Khaleghipour et al., 2021). A typical peak observed at 1080 cm^−1^ corresponds to C-O-C stretching. The other typical peaks of hemicellulose corresponding to 1739 and 1248 cm^−1^ in CW are observed, which refer to -COOH and ester functional groups, respectively (Rajinipriya et al., 2018). It can be seen that both these peaks disappeared for the AC-PT sample, indicating that hemicellulose was extracted from biomass successfully.

The thermogravimetric analysis of the CW and AC pretreated samples was performed; the findings are illustrated in Fig. 3(b)**.** The thermogram can be broadly categorized into three major phases. The temperature range 25–125 °C corresponds to a 5–10% loss in weight owing to moisture (Méndez-Durazno et al., 2025). The second major region is for hemicellulose breakdown (220–315 °C) (Ren et al., 2020) and decomposition of cellulose in the range of 330–400 °C (Phitsuwan et al., 2016). Above 500 °C, the flattened curve signifies the non-combustible fraction of biomass. For the AC-PT sample, changes can be observed. The cellulose combustion region was shifted towards the higher-temperature side of 360 °C. Also, the region referring to hemicellulose degradation is flattened as compared to the untreated one because of the successful extraction of hemicellulose by AC pretreatment. As hemicellulose is eliminated by pretreatment, the curve representing the degradation of hemicellulose becomes flattened.

The structure and crystallinity of untreated and pretreated biomass were studied by X-ray diffraction (XRD). Fig. 3 **(c)** shows peaks at 2θ = 16.07° and 22.1°, which can be assigned to the (100) and (002) cellulose I planes (Song et al., 2019). The AC-PT sample also exhibits similar peaks with minor 2θ changes and intensity. The CI, i.e. crystallinity index for CW (untreated), was 29.3%, and it increased to 30.5% for AC-PT. The pretreatment process removes amorphous hemicellulose, leading to exposure of crystalline cellulose. This supports successful hemicellulose/xylose extraction.

#### 3.1.4 CO_2_ emission and electrical energy consumption for AC-PT

The electrical energy consumption for AC-PT extraction of xylose was calculated (*P* = 2 kW; mass of CW = 40 mg; extraction duration = 20 min). Analysis of electric consumption for AC-PT is shown in Fig. 3(d)**.** For XOS production by enzymatic hydrolysis, the electric consumption was 0.666 kWh, and the relative consumption was 6.72 kWh/g xylose. Interestingly, the cauliflower consists of a very low amount of xylan (10–20% only), and by autoclave extraction and without any chemical treatment, 71.2% extraction of xylan from CW was obtained in a short duration of time. Thus, the process is green and quite energy efficient. During combustion, to produce 1 kWh of energy from fossil fuel, 800 g of CO_2_ is emitted into the atmospheric environment. The emission of CO_2_ during xylose extraction by AC-PT (*P* = 2 kW; CW = 40 mg; extraction duration = 20 min) is 0.546 kg. The method is efficient and environmentally friendly.

### 3.2 XOS extraction by enzymatic hydrolysis of AC-PT xylan

#### 3.2.1 Effect of BBD-based RSM optimization of EH for maximal XOS yield

To optimize the maximum XOS yields from AC-PT xylan via enzymatic hydrolysis, the impact of the three variables (temperature, enzyme loading and hydrolysis time) was optimized using RSM. There were a total of 17 runs of the experiment using the 3-factor-3-level BBD method. The levels of each component for BBD-based RSM are provided in the supplementary file (**Table S1)**. The hemicellulose fraction of the CW contained xylan, which was depolymerized by enzymes to provide a liquid extract with a significant amount of XOS (5.939 mg/mL), resulting in a conversion efficiency of 64.27% using xylanase. Subsequently, the yield of XOS (%) ranged from 35.48 to 64.27%, within 12–48 h of extraction time, enzyme loading of 15–35 U and a temperature of 40–60 °C. The highest XOS yield was 64.27%, observed with a temperature, hydrolysis duration, and enzyme loading of 50 °C, 30 h, and 25 U, respectively (Table 1).

**Table 1:** BBD examining the impact of various EH conditions on XOS yield.

**Run**	**EH**			**XOS (%)**	
	**EH1**	**EH2**	**EH3**	**Actual** Values*	**Predicted** **Values**
**1**	40	35	30	45.90	45.90
**2**	50	25	30	61.00	62.55
**3**	**50**	**25**	**30**	**64.27**	**62.55**
**4**	50	15	12	35.48	35.48
**5**	50	25	30	61.69	62.55
**6**	60	25	48	37.38	37.38
**7**	50	35	48	38.77	38.76
**8**	40	25	48	40.27	40.26
**9**	50	35	12	46.55	46.54
**10**	50	25	30	63.05	62.55
**11**	60	15	30	40.85	40.84
**12**	60	25	12	42.47	42.47
**13**	50	15	48	39.29	39.29
**14**	40	15	30	35.64	35.64
**15**	60	35	30	41.13	41.13
**16**	50	25	30	62.74	62.55
**17**	40	25	12	39.15	39.14

**EH1:** Temperature (°Celsius), **EH2:** Enzyme Loading (U) **EH3:** Hydrolysis Time (h) *mean ± SD (*n* = 3).

An ANOVA analysis of the experimental data produced the following quadratic polynomial equation:

Y_EH_ = 62.55 + 0.1087 A + 2.64B – 0.9925C - 2.50AB - 1.55 AC - 2.90 BCE - 10.94A^2^ -10.737B^2^–11.79C^2^.

Table 2 illustrates that the *p*-value (<0.0001) signifies the statistical significance of the model terms. The model's correctness and suitability were indicated by the coefficient of determination (R^2^ = 0.9966) and adjusted R^2^ (R^2^adj. = 0.9923) for XOS yield. The coefficient of variation (CV) of 2.04% signifies great precision and stability of experimental results, as well as substantial model fitting for the optimization of enzymatic hydrolysis to maximize XOS yield. The F-test indicates a substantial F-value of the model (F = 230.28) accompanied by a notably low *p*-value, highlighting a model fit that is statistically significant. The developed polynomial model demonstrated an exceptional fit, high predictive accuracy, and strong reliability.

**Table 2:** RSM-based ANOVA analysis demonstrating the effect of EH conditions on XOS.

**Source**	**Sum of Squares**	**df**	**Mean Square**	**F-value**	***p*-value**
**Model**	1890.95	9	210.11	230.28	< 0.0001^⁎⁎⁎^
A-Temperature	0.0946	1	0.0946	0.1037	0.7568^ns^
B-Enzyme Concentration	55.60	1	55.60	60.94	0.0001^⁎⁎⁎^
C-Hydrolysis Time	7.88	1	7.88	8.64	0.0217^⁎^
AB	24.90	1	24.90	27.29	0.0012^⁎⁎^
AC	9.64	1	9.64	10.57	0.0140^⁎^
BC	33.58	1	33.58	36.81	0.0005^⁎⁎⁎^
A^2^	503.70	1	503.70	552.08	< 0.0001^⁎⁎⁎^
B^2^	485.00	1	485.00	531.58	< 0.0001^⁎⁎⁎^
C^2^	585.78	1	585.78	642.04	< 0.0001^⁎⁎⁎^
**Residual**	6.39	7	0.9124		
Lack of Fit	0.0000	3	8.333	5.219	1.0000^ns^
Pure Error	6.39	4	1.60		
**Cor Total**	1897.34	16			
R^2^	0.9966				
Adjusted R^2^	0.9923				
Predicted R^2^	0.9947				
C·V	2.04%				

The Design-Expert software (version 13) produces 2D contour plots and 3D response surfaces that facilitate the analysis of interactive effects between two factors while keeping a third component constant.

**Fig. 4:**
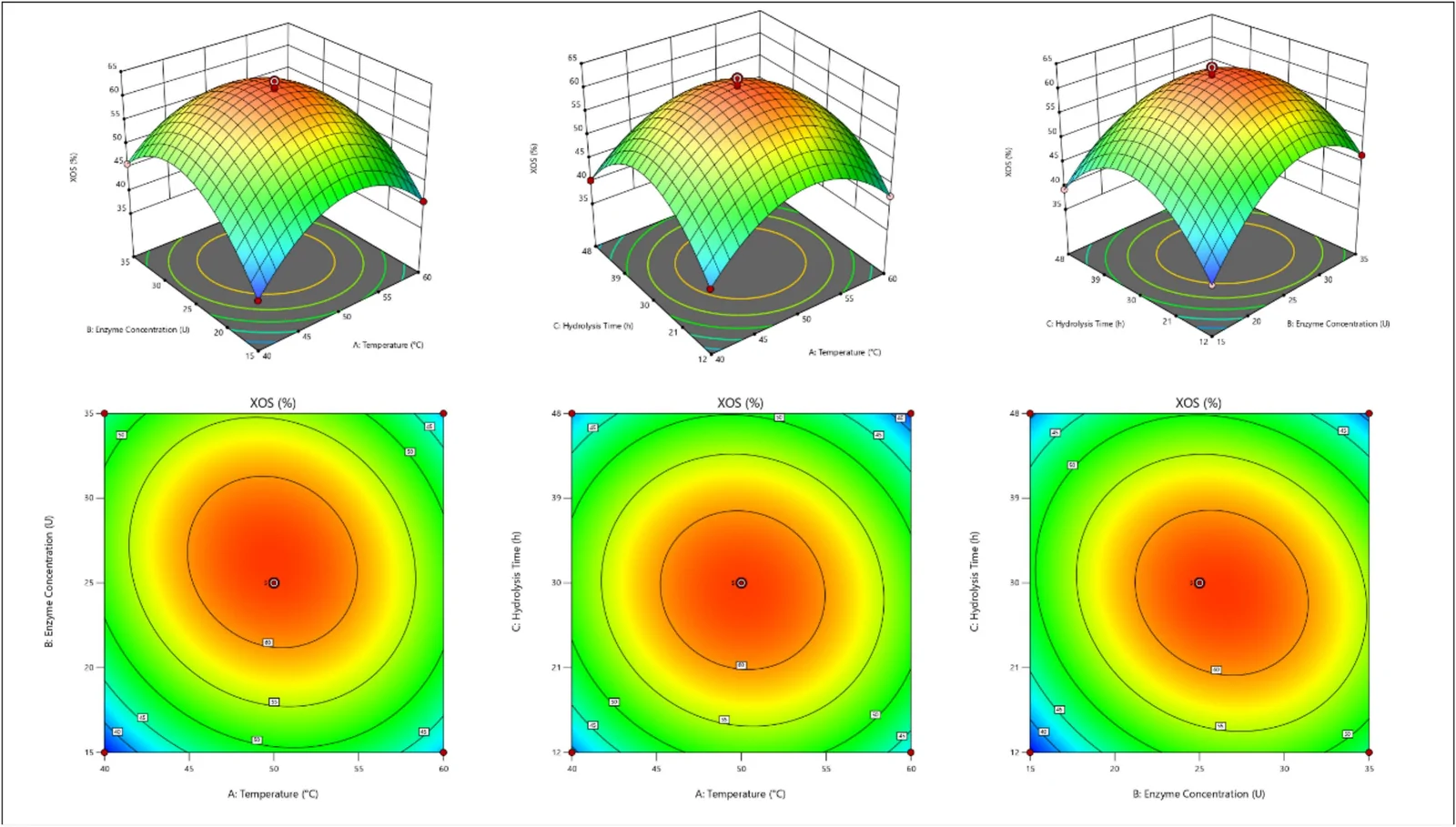
3D response surface and 2D contour plots for XOS production by EH.

Fig. 4 shows the 3D surface response and 2D contour plots generated from the BBD model for XOS production by enzymatic hydrolysis. Fig. 4 shows that as the temperature rises from 40 to 50 °C the XOS production increases at all enzymatic loadings, with the best performance of 64.27% at 25 U loading. The higher enzyme loading further decreases the yield, which may be due to increased hydrolysis leading to breakdown of XOS, as increased enzyme concentration could exceed the ideal threshold for substrate saturation and hence promote over-depolymerisation (Liao et al., 2022). The highest production was achieved at an optimal 50 °C. At lower temperatures, the low xylanase activity can limit the cleavage of the xylan backbone, and higher temperatures may lead to partial denaturation of the enzyme. The plot shows a strong quadratic effect of the variables temperature and enzyme loading. The quadratic temperature term (*p* < 0.0001) demonstrates its high significance and a nonlinear effect on XOS production. The XOS yield increased up to an optimum of 50 °C and then decreased at 60 °C, which may be attributed to the beneficial effect of increased enzymatic activity at moderate temperature being counteracted by reduced enzyme stability and/or secondary hydrolysis of XOS at higher temperature. The significant temperature × enzyme-loading (*p* = 0.0012) and temperature × hydrolysis-time (*p* = 0.0140) interactions also indicate that the effect of temperature was dependent on the other process variables. The highest XOS yield was observed at the highest enzyme dosage (35 U), within a shorter time duration, owing to a high hydrolysis rate. Increasing enzymatic load and prolonged hydrolysis duration generally lead to the secondary hydrolysis of XOS, leading to a decline. This observation is consistent with Michaelis-Menten kinetics, wherein the increase in enzyme loading increases the rate of reaction and reduces the time to attain the highest yield (Li et al., 2021). Hence, elevated enzyme concentration enhanced the initial hydrolysis, but decreased the XOS stability with prolonged incubation duration, making the 25 U at 30 min the best combination for high XOS production. The strong quadratic interactions are also exhibited in 2D contour plots. The robust interactions between enzyme loading-temperature, hydrolysis time-temperature and enzyme loading-hydrolysis time are depicted in the elliptical contour lines (Fig. 4). The hydrolysis time has a quadratic effect along with enzyme loading and temperature, making all of them significant parameters. The diagnostic plots for actual values vs predicted observations for XOS production by EH are in the supplementary file **(Fig. S1 (c))**. A high level of precision, i.e. 97.5% for RSM, was achieved for XOS production by enzymatic hydrolysis (for optimized conditions), showing excellent accordance with experimental results and strong predictive accuracy of the model.

#### 3.2.2 Purification and HPLC analysis for XOS: Efficiency

The HPLC analysis of XOS was carried out to quantify the % xylose conversion into XOS and short-chain xylo-oligosaccharides. The enzymatic hydrolysis of xylose by xylanase leads to the formation of XOS, resulting in a total concentration of 5.939 mg/mL of XOS. This represents a 64.27% conversion of xylose into XOS **(Fig. S2 (b)).** Among various fractions, short-chain XOS, specifically X2 (2.48 mg/mL), X3 (1.6096 mg/mL), and X4 (2.206 mg/mL), were present, with a small fraction of X6 (0.157 mg/mL). The percentage of fractions is as follows: X2 (38.4%), X3 (24.9%), X4 (34.3%), and X6 (2.4%). Therefore, the enzymatic hydrolysis of XOS largely results in the development of low DP XOS, as indicated by the substantial amount of X2-X4 (about 97.6%). When compared to monomeric xylose and high DP XOS, the generation of low DP XOS is advantageous for prebiotic and food applications due to their increased solubility and selective fermentation by beneficial gut bacteria, such as *Lactobacilli* and *Bifidobacterium* strains. Additionally, the resistance of low DP XOS to upper gastrointestinal digestion is a significant improvement. The literature provides extensive documentation of the functional benefits of short-chain XOS, which were previously discussed.

**Fig. 5:**
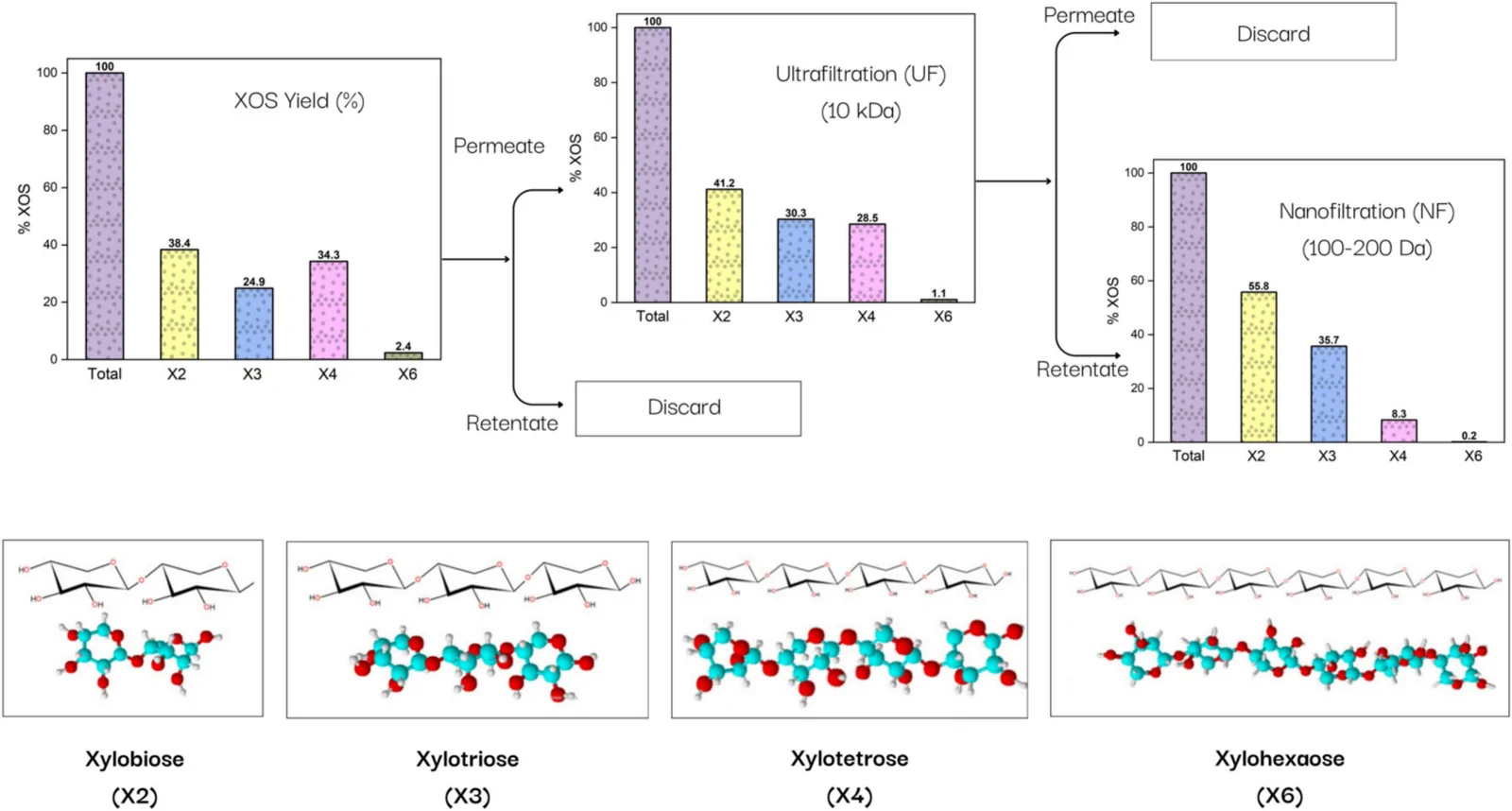
Membrane-based purification and fractionation of XOS using ultrafiltration and nanofiltration, showing changes in the relative distribution (%) of XOS fractions (X2-X6) at each purification step.

Following enzymatic hydrolysis, membrane-based fractionation utilizing ultrafiltration (UF) and nanofiltration (NF) was essential to concentrate low-degree polymerization XOS, while eliminating residual enzymes, proteins, monosaccharides, high molecular weight polysaccharides, and other contaminants from the hydrolysate (Sasaki et al., 2017). UF and NF sequential membrane systems efficiently enrich XOS from hydrolysates after enzymatic hydrolysis. As per the literature, the UF (10 kDa) could improve soluble carbohydrate recovery through controlled dilution with DW by removing unhydrolyzed xylan, lignin-rich macromolecules such as proteins and suspended particles in the retentate (Jiang et al., 2021), while allowing XOS to pass into the permeate. The last NF process (100–200 Da) can concentrate X2–X4, which are low-molecular-weight oligomers in the nanofiltration retentate, resulting in pure XOS fractions with reduced content of monosaccharides (Lehuedé et al., 2024) and other unwanted compounds, such as furfural and organic acids. This selective separation is in line with previous research showing that UF functions mainly as a pre-purification and clarification phase. By lowering membrane fouling and increasing permeate flux, this step aids in increasing downstream NF efficiency in the subsequent phase. Following UF, the permeate experiences partial enrichment of low-DP XOS and effective clarity. Consequently, the relative proportions of the X2 and X3 fractions grew to 41.2% and 30.3%, respectively, following UF. In contrast, X4 dropped to 28.5% and X6 to 1.1%. High molecular weight xylan residues, enzymes, and macromolecules were successfully eliminated after UF. The following nanofiltration further refined the XOS profile by selectively retaining oligosaccharides and may permit monosaccharide salts and contaminants to pass through the permeate. Consequently, the NF retentate exhibited significant enrichment of X2 (55.8%) and X3 (35.7%), alongside a notable decrease in higher DP fractions, including X4 (8.3%) and X6 (0.2%) **(**Fig. 5**).** The UF-NF system selectively purified and concentrated XOS with desired prebiotic potential for application in food and nutraceutical products, mainly X2-X3 fractions, which are reported to have enhanced fermentability and gut modification ability. Thus, the UF-NF system produced XOS from lignocellulosic hydrolysates without solvents, making it an efficient method for hemicellulose valorisation in sustainable biorefineries (Villar et al., 2026). The observed changes in the relative XOS distribution after UF–NF are consistent with molecular-weight-based fractionation and are supported by similar literature findings (Shi, Zhu, et al., 2025). However, direct analysis of the NF permeate would be required to conclusively confirm monosaccharide passage and individual XOS retention.

The MALDI-TOF MS data reveal a heterogeneous distribution of carbohydrate-associated ions, which are characteristic of XOS **(Fig. S3).** Multiple ions were detected in the *m*/*z* range 200–900, wherein the low- and medium-chain XOS dominate. The major chromatographic peaks were observed at retention times of 1.15, 1.87, 27.65, 34.27, and 39.86 min. Among these, the peak at 27.65 min represented the most abundant component. The analysis of peaks eluted at 27.69 min shows a dominant peak at *m*/*z* 282.311 along with isotopic ions at *m*/*z* 283.317 and 284.319. This ion is consistent with the molecular mass region for xylobiose (X2), suggesting low-degree polymerization XOS are predominant, which supports our HPLC findings. Deviations from ideal molecular weight at repeating intervals can be attributed to sodium/potassium adducts. The chromatographic peak at 33.32 and 34.87 min exhibited peak *m*/*z* = 406.36 and *m*/*z* = 407.37 for xylotriose (X3) together with ions at *m*/*z* 284.32(X2) and 310.33 (most probably [X2-Na]^+^ adduct.

The Na-associated carbohydrate ions are consistent with the literature that XOS predominantly form sodium adducts in positive ESI mode (Reis et al., 2004). The peak at *m*/*z* = 569.36 corresponds to X4 and is consistent with the [X4-Na]^+^ adduct. The results are supported by the literature (Brasseur et al., 2014; Reis et al., 2004). The ions observed at *m*/*z* 885.6–885.7 can be assigned to higher-degree polymerization xylooligosaccharides as X6, mostly as alkali-metal adducts or substituted derivatives. The lower abundance of high DP ions compared to X2 or X3 indicates that the hydrolysis process preferentially generated low molecular weight XOS. Such distributions are commonly observed following endoxylanase-mediated hydrolysis, where xylobiose and xylotriose constitute the principal products while higher oligomers are present in smaller quantities. The MS analysis supports that XOS mainly contains low DP, with X2 and X3 as major components, with lower amounts of X4 and X6.

Further MS analysis was again performed for purified XOS powder obtained after the ultrafiltration-nanofiltration sequential route **(Fig. S4).** The reduced spectral complexity relative to crude XOS indicates successful purification and enrichment of low-degree polymerization XOS species. The LC–ESI–TOF–MS spectra of the purified XOS fraction (retention times 26.45, 26.49, and 33.41 min) demonstrate a highly simplified oligosaccharide profile with X2 and X3 as the dominant ones as compared with the crude XOS sample, indicating effective purification by ultrafiltration (UF) and nanofiltration (NF). The predominance of a few intense peaks and the absence of high-molecular-weight ions suggest successful removal of polysaccharides, lignin-derived compounds, salts, and other low-molecular-weight impurities.

Consistent with the circular bioeconomy principles, xylan derived from cauliflower waste serves as a substrate for the manufacturing of enzymatic XOS that is both cost-effective and sustainable. This is accomplished by converting low-value waste into a high-value ingredient. The process follows a logical sequence: cauliflower waste autoclave hydrothermal pretreatment followed by centrifugation/clarification, enzymatic hydrolysis and ultra/nanopurification to get purified XOS. For scale-up and industrial considerations, the batch-wise nature of autoclave hydrothermal treatment and the chances of membrane fouling during UF–NF are important issues. To address these issues, future process improvements should investigate continuous or semi-continuous autoclave-hydrothermal reactors. In addition, the pre-treatment of the hydrolysate by clarification and solid-liquid separation could reduce membrane fouling by decreasing the suspended solids and macromolecular impurities that may enter the membrane. The operational stability and performance of the membrane can be improved by optimisation of transmembrane pressure, cross-flow conditions, membrane configuration and periodic membrane cleaning/regeneration. The amalgamation of these strategies along with process monitoring and mass-balance analysis would surely enhance the filtration performance and support the technical and economic viability of large-scale production of XOS from cauliflower waste.

### 3.3 Mass balance and cost analysis

The integrated autoclave-hydrothermal pretreatment, xylose extraction, enzymatic hydrolysis and filtration process effectively redistributed the biomass, which supported the selective production of high-value low DP XOS. Beginning with 1 g of cauliflower waste powder, 138.60 mg xylose was recovered, compared to the theoretical 193.18 mg xylose availability (17% in cauliflower), leading to a 71.75% extraction efficiency by autoclave-hydrothermal pretreatment. Subsequently, the enzymatic hydrolysis yielded 89.09 mg XOS, which accounted for an apparent 64.27% conversion. The sequential UF–NF filtration recovered nearly 75.70 mg purified XOS, i.e. 84.98% membrane recovery. The final purified product, i.e. XOS (69.27 mg), contained mainly low-DP X2 and X3, which accounted for 91.5% of the purified product. The unrecovered fraction may be explained by the associated membrane losses, permeate losses, or retention of XOS in intermediate streams.

The cost analysis for high-quality XOS production from CW biomass via sequential AC-hydrothermal pretreatment-enzymatic hydrolysis-filtration/purification was performed to assess the economic feasibility of the strategy (**Table S4**). The total approximate cost for XOS production from 0.050 Kg CW biomass, accounting for energy cost for processing, membrane filtration, and enzymatic hydrolysis, was calculated as Rs. 1535. The approximate cost for producing 1 g XOS was estimated as Rs. 345. The membrane-related costs have been included for cost assessment based purely on the purchase cost of the membranes. At larger/industrial scale, reusable TFF membranes could lower the membrane cost per gram of XOS on a wider scale by using several approved manufacturing cycles. Since this cost estimation is only based on laboratory-scale XOS production, the cost for XOS/g will reduce significantly on scale-up. This economic feasibility analysis shows that the sequential process is technically feasible, especially for low DP (with superior prebiotic activity) XOS for high-quality prebiotic food products from a vegetable waste feed. For scale-up, enzymatic hydrolysis may be carried out in bigger stirred-tank reactors under regulated pH and temperature settings, while continuous hydrothermal processing with heat recovery could take the place of batch hydrothermal pretreatment. The techno-economic analysis has also shown that process economics can be significantly enhanced by raising XOS production capacity and valuing coproducts.

### 3.4 Toxicity analysis

#### 3.4.1 Plant cytotoxicity

**Fig. 6:**
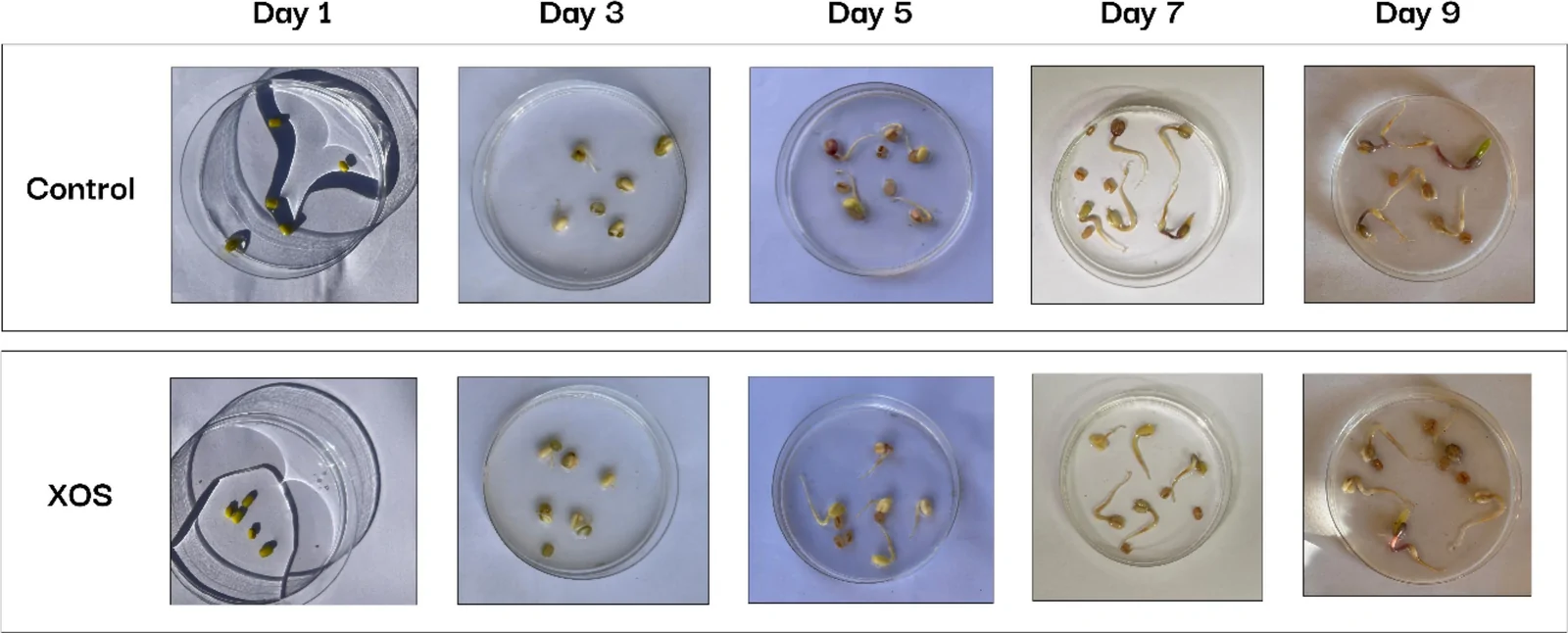
Plant cytotoxicity evaluation of XOS using the mung bean (*Vigna radiata*) germination assay. Seed germination and seedling growth in (a) control (deionized water) and (b) XOS-treated groups were observed on 1, 3, 5, 7 and 9 days, indicating the absence of phytotoxic effects of XOS.

The plant cytotoxicity was tested by conducting mung bean germination and growth studies (Chen et al., 2025), and the results are shown in Fig. 6 for 1st, 3rd, 5th, 7th, and 9th days. The XOS-treated group and the group that was treated only with DW both showed the development of healthy green sprouts with established roots and stems. The group that received the XOS sample treatment showed robust leaf growth and somewhat tall plants on day nine. The data indicate that XOS might be regarded as non-toxic.

#### 3.4.2 Cytotoxicity analysis on human cell line

**Fig. 7:**
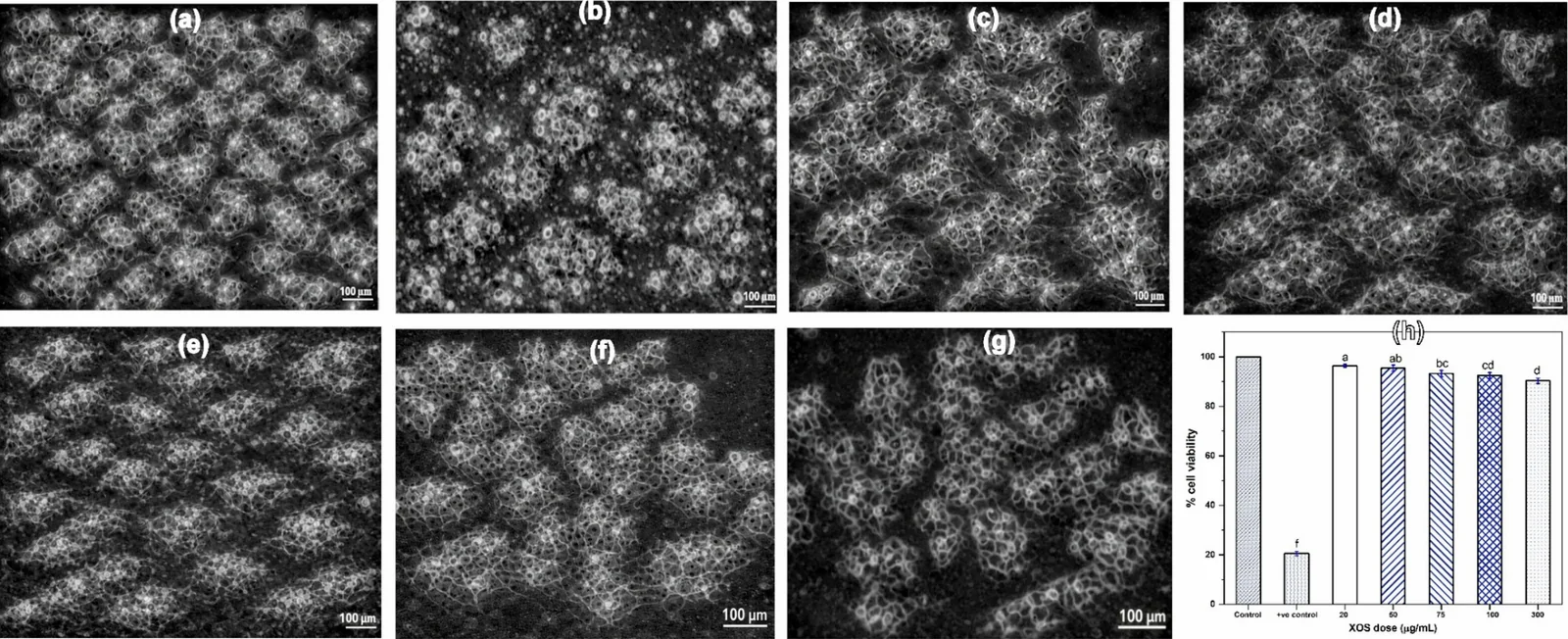
In-vitro cytotoxicity of XOS on NCM460 human normal colon epithelial cells using the MTT assay. Representative fluorescence microscopy images of cells treated with (a-b) negative and positive controls, (c-g) variable XOS doses; (h) Cell viability (%) in relation to the untreated counterpart. The values (*n* = 3 separate experiments) are expressed as mean ± SD. Statistical analysis was conducted using one-way ANOVA and Tukey's multiple-comparison analysis. Significant differences between the groups are indicated through distinct lowercase letters above the bars (*p* < 0.05).

Cytotoxicity of XOS on the NCM460 cell line was checked in a concentration-dependent manner by employing the standard MTT assay methodology (Edmondson et al., 1988). To investigate whether XOS affect cell viability, cells were treated with XOS (20–300 μg/mL) for 24 h. These results (Fig. 7a-g**)** showed that none of the chosen XOS doses utilized in the subsequent investigation produced cytotoxicity in the NCM 460 monolayers after a 24-h incubation period. Also, XOS have been reported to possess immunomodulatory activity (Aachary & Prapulla, 2011).

The cells' activity did not change much after being exposed to XOS for 24 h at a dose of 20–300 μg/mL. The cell viability results are shown in Fig. 7 **(h).** For a 20 μg/mL concentration, the cell viability was as high as 97.1 ± 1.34%, and high viability was also obtained for the highest XOS concentration of 300 μg/mL. For the positive control, Triton X-100, the cell viability was 20.6 ± 1.23%. For a higher concentration of XOS, the viability of the cells was reduced slightly. No abnormal reduction in cell growth was reported. Microscopic observation revealed that the reduction in cell number caused by XOS was due to the inhibition of cell proliferation rather than the induction of cell death. The lactate dehydrogenase (LDH) levels in the medium of each experimental group did not, however, change significantly within the measured concentration, suggesting that the structural integrity of the cell membrane was preserved.

### 3.5 *Antioxidant activity of XOS*

The antioxidant activity of xylan (obtained from autoclave-assisted extraction from CW) and XOS obtained by EH of xylan was measured by the DPPH method. The results indicate that XOS exhibit higher antioxidant activity (11.64%) than xylan (7.43%) itself. When compared with the CW (raw), which had an antioxidant activity of 51.81%, the xylan and XOS showed a relatively low level of antioxidant activity. XOS functional characteristics are largely affected by their degree of polymerization and degree of substitution (Wu et al., 2020). According to (Valls et al., 2018), high-DP XOS are better antioxidants. It was reported that after filtering XOS from guava seed cake, DPPH scavenging did not exceed 0.5%, compared to 84.2% without filtering (Forsan, Marin, & Brienzo, 2025). The purification of XOS could have removed phenolic substances, aromatic compounds, lignin derivatives, and degradation products from XOS. It has been found in the literature that xylans have limited antioxidant activity in-vitro (da Cruz Filho et al., 2023). Xylan showed 10% activity in the DPPH radical capture assay due to lower concentrations of phenolic compounds.

### 3.6 Antimicrobial properties of XOS

**Fig. 8:**
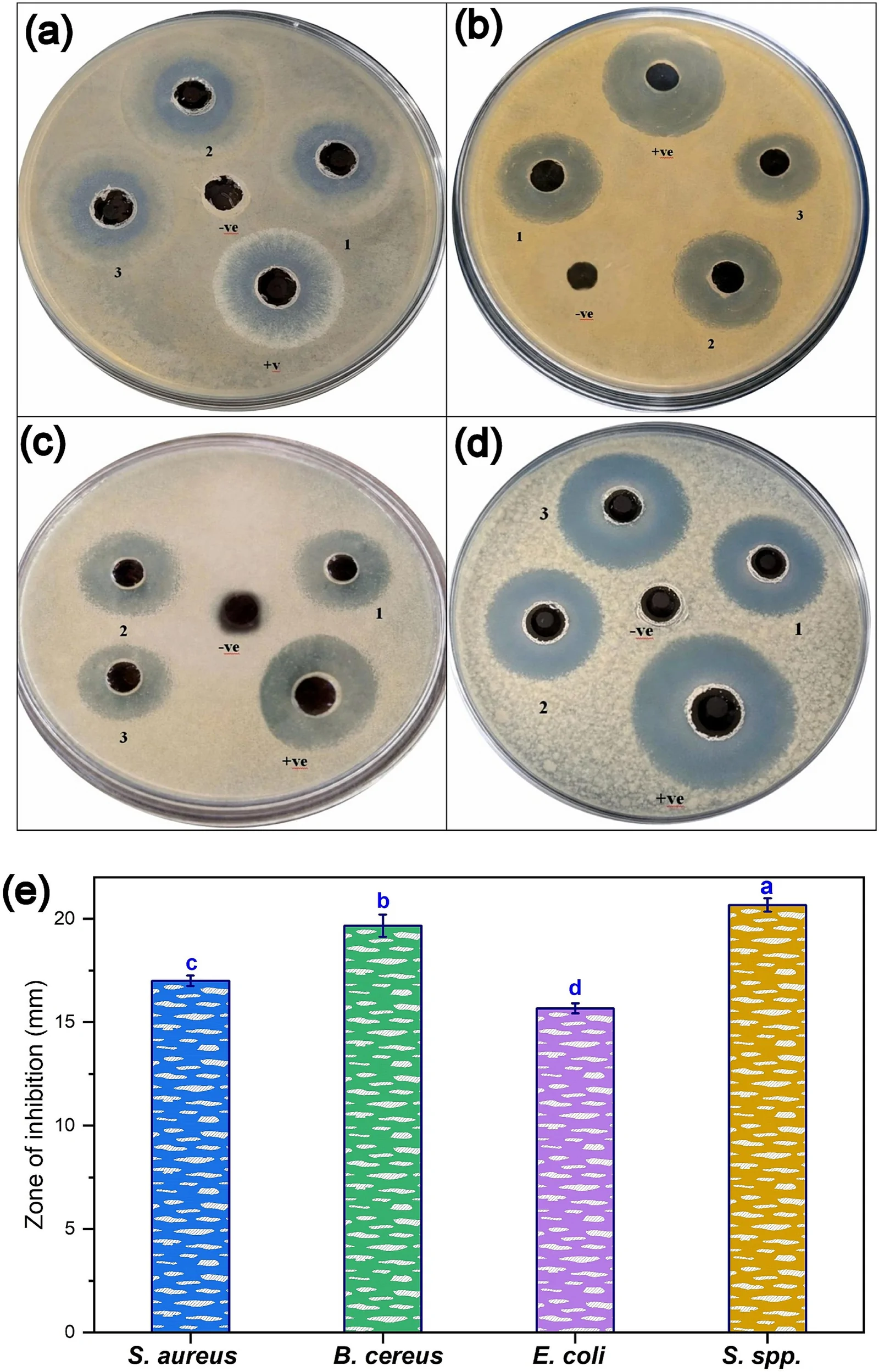
Agar diffusion assay showing the antimicrobial activity of XOS: (a) *S. aureus,* (b) *B. cereus,* (c) *E. coli,* and (d) *S.* spp.*,* (e) corresponding zone of inhibition diameters (mm). Values are expressed as mean ± standard deviation (*n* = 3 independent experiments). One-way ANOVA and Tukey's multiple comparison test were used for statistical analysis. Distinct lowercase letters above bars indicate significant group differences (*p* < 0.05).

The XOS demonstrated measurable inhibitory activity against all tested microorganisms, indicating a broad-spectrum antimicrobial potential **(**Fig. 8a-d**)**. For Gram-positive bacteria, XOS exhibited a mean zone of inhibition of 17.00 ± 1.73 mm and 19.67 ± 1.15 mm for *Staphylococcus aureus* and *Bacillus cereus,* respectively. Although the inhibitory activity was less pronounced when observed for the positive control (gentamicin; 26 mm), the clear and reproducible zones confirm the antibacterial efficacy of the test fraction (Fig. 8e**)**. This indicates a stronger antibacterial effect of the XOS against this organism, which may be attributed to the relatively permeable thick peptidoglycan-rich cell layer of Gram-positive bacteria. Further, for Gram-negative bacteria, *E. coli* and *S.* spp. exhibited ZOI of 15.67 ± 2.31 mm and 20.67 ± 1.15 mm, respectively. In the sterile distilled water negative control wells, there was no zone of inhibition, confirming that the observed antimicrobial effects were solely attributable to the test sample. XOS exhibited slightly stronger activity against Gram-positive organisms overall, a trend commonly reported for plant- derived or carbohydrate-based antimicrobial compounds.

### 3.7 Prebiotic potential analysis of XOS and digestibility

**Fig. 9:**
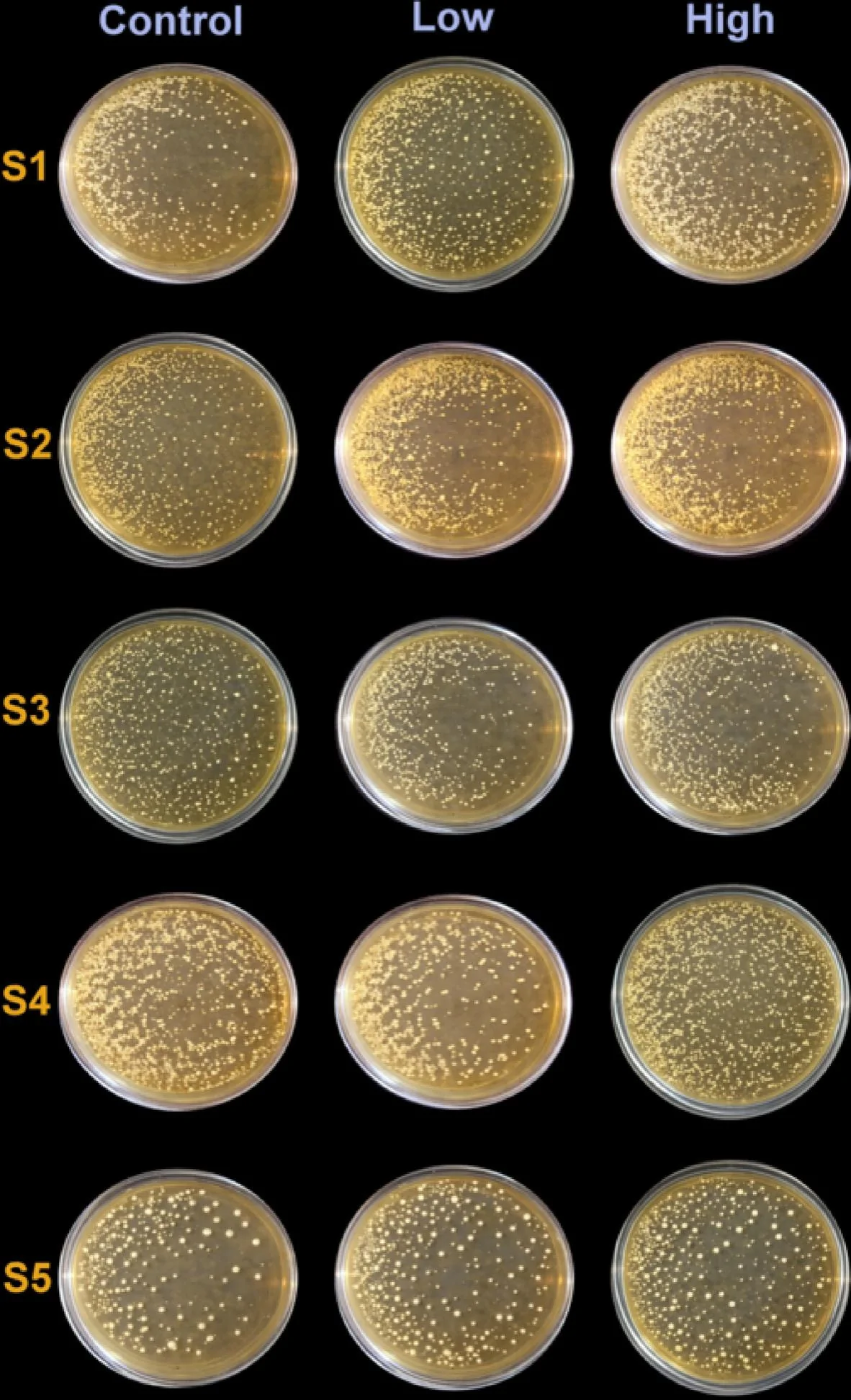
In-vitro prebiotic potential assessment of XOS by fermentation assay: Representative colony growth of probiotic strains (S1-S5) on modified MRS agar supplemented with control (glucose) and XOS at low (1 mg/mL) and high concentrations (2 mg/mL) after 24 h incubation.

**Fig. 10:**
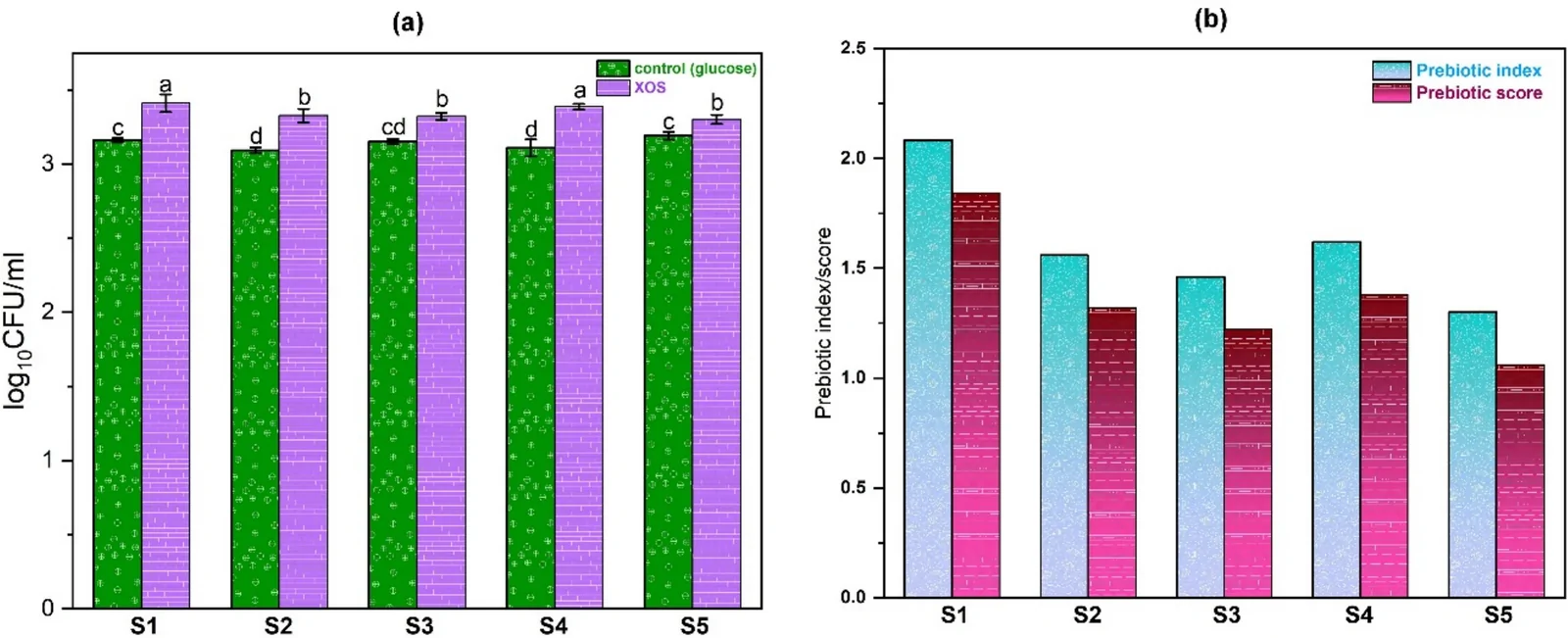
In-vitro prebiotic potential assessment of XOS by fermentation assay (a) Growth expressed as log_10_ CFU/mL; each value on the bar represents mean ± SD. (b) PI and PS of XOS based on probiotic growth in beneficial bacteria relative to the control carbohydrate and suppression of *E. coli.* Values are reported as mean ± SD (*n* = 3). Significant variation between treatments was tested employing two-way ANOVA, after Tukey's multiple comparative assessment, with distinct lowercase letters indicating statistically significant variances (*p* < 0.05).

The five strains *Lactobacillus plantarum* (S1), *Lactobacillus fermentum* (S2)*, Lactobacillus brevis* (S3), *Lactobacillus rhamnosus* (S4) and *Lactobacillus casei* (S5) were used to test the prebiotic potential of the purified XOS. Fig. 9 shows the colony growth of probiotic strains after 24 h incubation in XOS (low conc-1 mg/mL and high conc-2 mg/mL). Glucose was employed as a conventional positive control because it possesses faster metabolism and association. In general, a progressive increase in cell proliferation over time for all probiotic strains was observed at lower as well as higher concentrations of XOS (Fig. 9) and was higher than glucose for all strains. The growth was higher with XOS than with the control, i.e., glucose, for all *Lactobacillus* strains. The proliferation follows the order according to Log_10_CFU/mL values: S1 (3.44) > S4 (3.39) > S3 (3.33) > S2 (3.31) > S5 (3.30) (shown in Fig. 10a). These observations establish the extracted XOS from CW as a substrate promoting the growth of useful *Lactobacillus* species. The xylooligosaccharides have a proven record of superior prebiotic and antioxidant activity (Chaiyates et al., 2025). During XOS fermentation by the beneficial bacteria, SCFAs are formed, which leads to improvement in the immune system and regulation of antibody production (Forsan, Ávila, et al., 2025). Because of their oligomeric nature, XOS are slowly digested and maintain probiotic development for longer. However, glucose (monomeric) is digested rapidly and cannot sustain development. The differential responses obtained across *Lactobacillus* species show the important role of carbohydrate-active gene content in desired and efficient prebiotic fermentation results. The purified form of XOS has a higher proportion of X2-X4, which is a quality standard because they are major bioactives. The growth-enhancing effect of XOS probiotics is ascribed to the activation of several genes that govern cellular division and the synthesis of cellular constituents, hence affecting glucose and ribosome metabolism (Shi, Wang, et al., 2025).

The results here show that XOS is well utilized and hence supports probiotic proliferation, with the best results for L. *plantarum* and L. *rhamnosus*. It has been reported from transcriptomic analysis that L. *plantarum* would yield numerous differentially expressed genes during the XOS fermentation, especially the RS03575 transporter peptide, which could mediate the import of XOS into the cell (Y. Shi et al., 2025). *L. plantarum* also expresses β-xylosidase activity that facilitates XOS breakdown, supporting growth and energy production. *L. rhamnosus* is also considered to carry numerous carbohydrate transport and utilization genes, which may enhance the carbohydrates utilization (Douillard et al., 2013). It was reported earlier that *Lactobacilli* strain*s* have the capability of growing in XOS-containing media and producing lactic acid and short-chain fatty acids (Iliev et al., 2020). These observations are supported by the literature (Ceapa et al., 2015; Cui et al., 2021). Especially in L. *plantarum* and L. *rhamnosus,* XOS not only acts as a substrate but also leads to upregulation of genes that are related to ribosomal biogenesis and carbohydrate metabolism, which leads to better adaptation in comparison to other strains and glucose itself. Alternative strains, such as L. *casei*, L. *fermentum* and *L. brevis,* grow on purified XOS but to a lower extent than the other two strains, owing to the fact that they may have fewer or less specific transporters. The ability of certain *Lactobacillus* species to preferentially metabolize XOS is also rooted in their carbohydrate uptake systems and enzymatic pathways. This good probiotic growth is notable because it is associated with enhanced gut barrier function and immune modulation, which helps in the promotion of overall gut health. It was additionally noted that the proliferation of possibly hazardous microorganisms, *E. coli,* decreased over time, suggesting that XOS also suppresses these bacteria.

The prebiotic index (PI) measures the growth comparison of probiotic bacteria (by prebiotic utilization, XOS in this case) in MRS medium with glucose (control) as a C-source in a 24 h incubation. Values below one indicate low efficiency of the prebiotics used for probiotic utilization. The prebiotic index (Fig. 10b), as calculated, follows the order: S1 (2.083) > S4 (1.62) > S2 (1.56) > S3 (1.46) > S5 (1.3). All the probiotics show high PI values, suggesting good prebiotic activity, with the best performance for L. *plantarum.* The purified XOS thus exhibited a stimulating impact on growth as compared to the glucose control. Further, the prebiotic score was calculated via comparison of the difference in growth between probiotic bacteria with glucose and in the media with prebiotic and *E. coli* (reference bacteria). A prebiotic activity score below one indicates that *E. coli* predominates over beneficial probiotic bacteria, while a score over one denotes the inhibition of potentially hazardous bacteria by probiotic bacteria utilizing prebiotics (specifically XOS) as a carbon source. In this work, a prebiotic activity score > 1 was obtained for all bacteria, with L. *plantarum* and L. *rhamnosus* having the highest scores of 1.844 and 1381. The in-vitro prebiotic assessment in this work included glucose as a positive carbohydrate (C-source) control, no‑carbon-source as a negative control, for five microbial strains and *E. coli* as a reference organism. Negligible growth was obtained in the negative control. Nevertheless, future work could further strengthen the prebiotic evaluation using standard reference prebiotics.

**Fig. 11:**
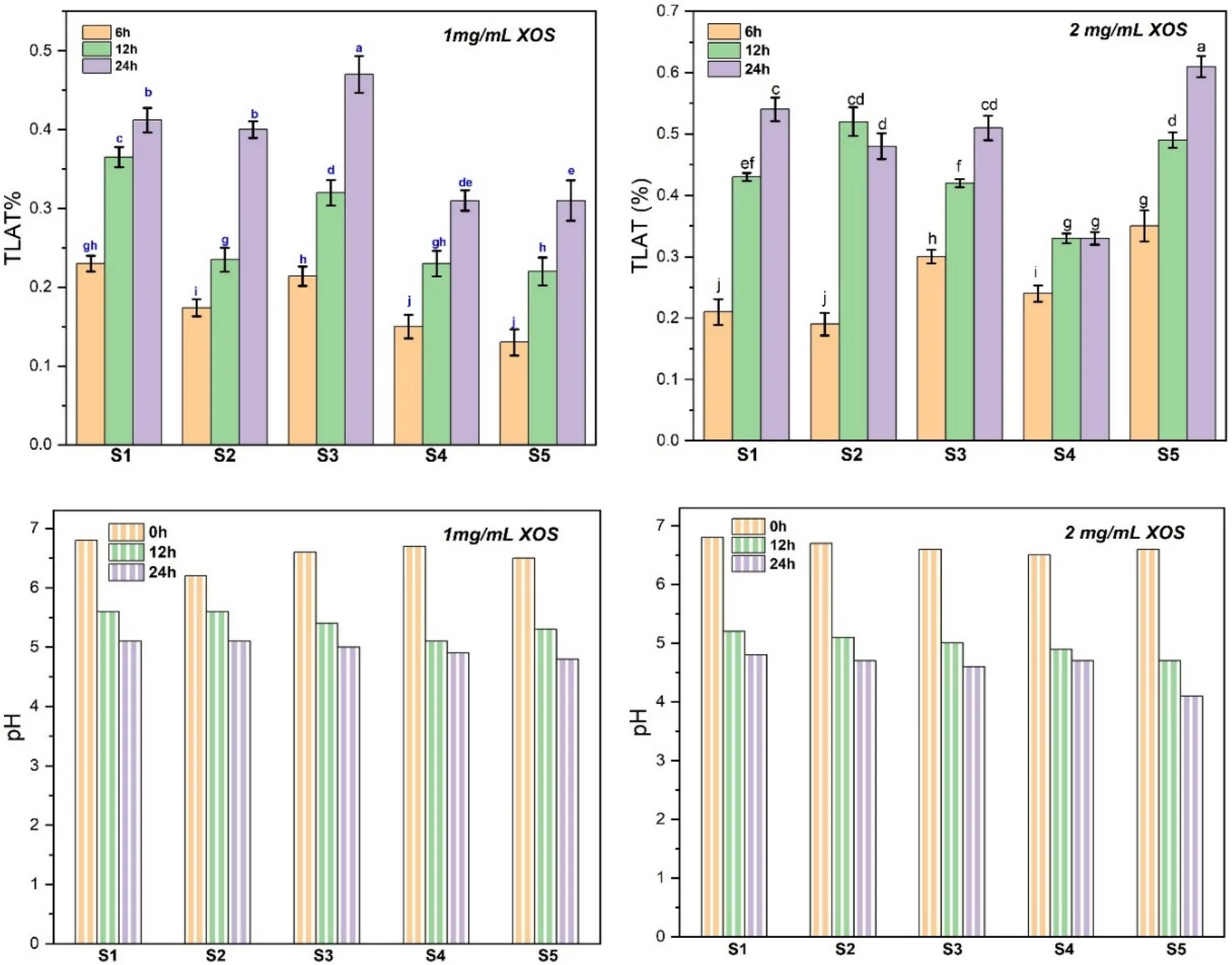
Total lactic acid titrated (TLAT) for fermented media (treated with XOS) (a) 1 mg/mL (b) 2 mg/mL. Each value on the bar represents the mean ± SD. The pH changes for fermented media (treated with XOS) were (c) 1 mg/mL (d) 2 mg/mL. Values are presented as mean ± SD (*n* = 3). Significant variation between treatments was tested using two-way ANOVA, after Tukey's multiple comparative assessment, with distinct lowercase letters indicating statistically significant variances (*p* < 0.05).

The fermentation of XOS by bacteria produces lactic acid and other SCFAs. Fig. 11 **(a-b)** shows results for total lactic acid titrated (TLAT) from medium containing low (1 mg/mL) and high (2 mg/mL) purified XOS from cauliflower waste powder. Based on the graph, TLAT increased significantly until 24 h for both concentrations. It was nearly 0.23% at 6 h for L. *plantarum,* but then it increased rapidly to 0.41% in 24 h for the low XOS concentration. However, it reached 0.54% over 24 h in media with a 2 mg/mL XOS concentration. In 24 h, media with 2 mg/mL XOS exhibited 0.51% total lactic acid for L. *rhamnosus* and 0.61% for L. *casei*. The results are inconsistent at low concentration but more consistent at higher XOS concentration. The TLAT is direct metabolic evidence of XOS utilization, not just growth stimulation. As discussed above, the transporters import XOS into the cell, wherein they are hydrolysed and enters further into the phosphoketolase pathway, leading to lactic acid production and further short-chain fatty acids. Fig. 11 **(c-d)** illustrates a decline in the pH of the fluid during the incubation period. The pH value for media supplemented with 2 mg/mL XOS for L. *plantarum* decreased from 6.8 to 4.8 in 24 h. For *Lactobacillus fermentum, Lactobacillus brevis, Lactobacillus rhamnosus* and *Lactobacillus casei,* the pH decreased to 4.7, 4.6, 4.7 and 4.1, respectively, in 24 h. The literature supports these findings (Ratnadewi et al., 2020). There are some irregular changes in pH, which may be due to the formation of ammonia by the breakdown of amino acids from the media (Klangpetch et al., 2022). The lactate intermediate gets gradually converted into several SCFAs such as acetate, propionate and butyrate (Belenguer et al., 2006). The SCFAs were further analyzed by GC-FID. Acetic acid, detected as acetate, was produced at high concentration in all the assays (for all fermentation assays from all five bacteria), comprising more than 90% of the obtained extracts. Acetic acid (acetate) helps in reducing the intestinal pH, regulating blood pH regulation and inhibiting the development of pathogenic bacteria (Holmes et al., 2020). The second most abundant SCFA produced is propionate. The production of SCFAs during in-vitro fermentation of XOS with probiotics is important, as these fight against inflammation and lower the level of bad cholesterol, i.e., LDL levels. Additionally, butyrate was also confirmed in all assays. Butyrate is important as it reduces intestinal inflammation. The production and distribution of SCFAs following fermentation by probiotic strains S1, S2, S3, S4, and S5 are presented in **Table S5.** High SCFA production was achieved for S1, S3 and S4 and low for S2 and S5 strains. The total SCFA concentration ranged from 2.10 to 2.48 mg/mL, indicating efficient utilization of XOS by all strains. Among the tested strains, S4 produced the highest total SCFA concentration (2.48 ± 0.11 mg/mL), followed by S1 (2.22 ± 0.31 mg/mL) and S3 (2.10 ± 0.23 mg/mL), suggesting superior fermentative activity of strain S4. The elevated SCFA production observed in the present study confirms the prebiotic potential of XOS and its ability to stimulate beneficial microbial metabolism. Acetic acid was the predominant SCFA produced by all strains, with concentrations ranging from 1.99 to 2.25 mg/mL, accounting for 90.73–94.76% of total SCFAs. Overall, the SCFA profile generated by all strains was characterized by a high proportion of acetate (>90%), moderate levels of propionate (3.81–6.45%), and lower but physiologically relevant levels of butyrate (1.43–2.25%). Branched-chain fatty acids were detected only in trace amounts.

**Fig. 12:**
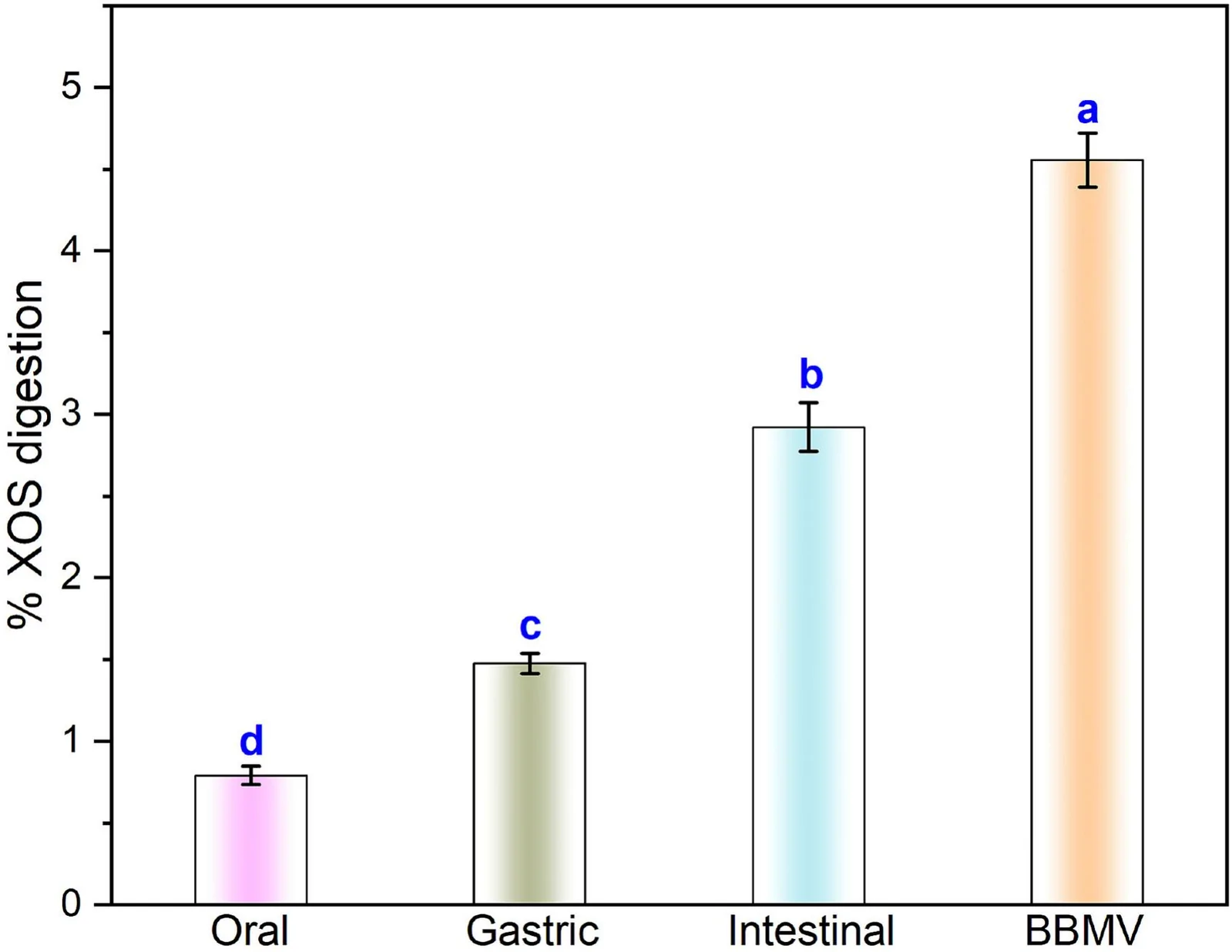
Percentage digestion of XOS during simulated oral, gastric, intestinal, and BBMV digestion stages. Data are expressed as mean ± SD (*n* = 3). Different lowercase letters indicate significant differences among treatments (Tukey test, *p* < 0.05).

To evaluate how resistant the purified XOS is to enzymes involved in the digestive process, in-vitro digestibility tests were further performed that modelled the various stages of digestion: the oral phase, the gastric phase, the intestinal phase, and the Brush Border Membrane Vesicles (BBMV) phase (Fig. 12). The data (**Table S6**) show only 9.72% of total digestibility for XOS, indicating that the majority (90.28%) of XOS remained intact following the different simulated stages (oral, gastric, intestinal and BBMV phases). The smaller oligosaccharides and α-limit dextrins cannot be digested by α-amylase (Klangpetch et al., 2022). The purified XOS contains only low-DP ones and thus is poorly digested in the oral phase (0.78%). A 1.47% degree of digestion was obtained during the gastric phase, as the stomach enzymatically hydrolyses the XOS through HCl (Klangpetch et al., 2022), and 97.75% of XOS still remains intact. Further, 2.92% of the digestibility was measured during the intestinal phase. The BBMV phase exhibited a higher 4.55% digestibility of XOS. This is because the brush border membrane vesicle enzyme is the last step in the process of carbohydrate digestion in the small intestine. However, the total digestibility was still less than 9.72%, and 90.28% of the XOS remained undigested, indicating a high level of resistance of XOS to gastrointestinal digestion (da Silva et al., 2024). The XOS, thus, will reach the colon and provide a fermentable substrate for several gut microbes. These results satisfy the necessary fundamental criteria for the XOS to be labelled as prebiotic oligosaccharides.

## 4 Conclusions

This work reports the valorisation of cauliflower waste into xylose and further xylo-oligosaccharides by sustainable sequential autoclave hydrothermal pretreatment-enzymatic hydrolysis-membrane filtration as a potential prebiotic nutraceutical. The autoclave-assisted extraction of xylose from cauliflower waste (underutilized and poorly valorised) leads to 71.7% xylose extraction efficiency. Both the processes of pretreatment for xylan extraction and xylanase-assisted enzymatic hydrolysis for XOS production were optimized for high yield by RSM using BBD with excellent model fitting. One of the main highlights was the achievement of a substantial 97.6% low DP XOS, i.e., X2- X4, and following ultrafiltration and nanofiltration, the XOS purity was concentrated to 55.8% X2 and 35.7% X3, with decreased high-DP fractions. The lower DP fractions possess higher prebiotic activity. The aquatic toxicity tests conducted on mung beans and NCM 460 human cell lines revealed no toxicity. The in-vitro fermentation assay results revealed that XOS was well utilized and hence supports probiotic proliferation with high selectivity for L. *plantarum* and L. *rhamnosus.* The good probiotic growth is highly promising as it would improve gut barrier function and immune modulation. The purified XOS fosters the proliferation of advantageous microorganisms and suppresses harmful bacteria, such as *E. coli*. Decreased over time, suggesting that XOS also suppresses these bacteria. In conclusion, the production of XOS from food wastes by green technologies can be economic and ecological, as it allows sustainable bioconversion of recalcitrant lignocellulosic biomass into prebiotic-based healthy foods.

## CRediT authorship contribution statement

**Piyush Verma:** Writing – original draft, Methodology, Investigation, Formal analysis, Data curation, Conceptualization. **Amit Kumar:** Writing – review & editing, Validation, Supervision, Methodology, Conceptualization. **Ravinder Kaushik:** Writing – review & editing, Validation, Supervision, Conceptualization. **Pooja Dhiman:** Writing – review & editing, Visualization. **Chuanling Si:** Writing – review & editing, Validation. **Chin Wei Lai:** Writing – review & editing, Resources.

## Declaration of competing interest

The authors declare that they have no known competing financial interests or personal relationships that could have appeared to influence the work reported in this paper.

## Data Availability

Data will be made available on request.

## Appendix A Supplementary data

Supplementary material BBD design parameters; Experimental-MS analysis, prebiotic simulation, energy consumption, plant toxicity, cyto-toxicity, antimicrobial and antioxidant properties; BBD analysis; HPLC chromatograms; LC-MS spectra; Cost analysis and prebiotic digestibility.
