# Supplementary material for: Sequential autoclave–hydrothermal, enzymatic conversion of cauliflower waste Xylan into high-purity bioactive Xylooligosaccharides: Purification, prebiotic activity and toxicity analysis
Source: Food Chem X. 2026 Sep 10;39:104428. doi: 10.1016/j.fochx.2026.104428 (PMC13594635; doi:10.1016/j.fochx.2026.104428)
Supplement: Supplementary file 1 [file mmc1.docx]

**Electronic Supplementary Files**

**Sequential Autoclave–Hydrothermal, Enzymatic Conversion of Cauliflower Waste Xylan into High-Purity Bioactive Xylooligosaccharides: Purification, Prebiotic Activity and Toxicity Analysis**

Piyush Verma^a^, Amit Kumar^a,e*^, Ravinder Kaushik^a*^, Pooja Dhiman^c^, Chuanling Si^d^, Chin Wei Lai^b*^

*^a^School of Health Sciences and Technology, UPES, Dehradun, Uttarakhand, 248007, India*

*^b^Nanotechnology & Catalysis Research Centre (NANOCAT), Institute for Advanced Studies*

*(IAS), University of Malaya (UM), 50603 Kuala Lumpur, Malaysia*

*^c^International Research Centre of Nanotechnology for Himalayan Sustainability (IRCNHS), Shoolini University, Solan, 173229, India*

*^d^State Key Laboratory of Bio-Based Fiber Materials, Tianjin Key Laboratory of Pulp and Paper, Tianjin University of Science and Technology, Tianjin, 300457, People’s Republic of China*

*^e^Faculty of Engineering and Technology, Sunway University, Jalan Universiti, Bandar Sunway, Selangor, Petaling Jaya, 47500, Malaysia*

**Corresponding author:* [*mittuchem83@gmail.com*](mailto:mittuchem83@gmail.com) (A. Kumar), [ravinderkaushik@ddn.upes.ac.in](mailto:ravinderkaushik@ddn.upes.ac.in) (R. Kaushik), [cwlai@um.edu.my](mailto:cwlai@um.edu.my) (C.W. Lai)

*S2.1 BBD-based statistical experimental design*

A three-factor BBD was used to study the response trend and identify the best combination of operational parameters/factors for maximizing sugar output or hemicellulose/xylan extraction. According to the literature (Dhaver, Pletschke, Sithole, & Govinden, 2023; Kathiresan, Karuppiah, Gopal, Abraham, & Thangavel, 2025; Su et al., 2021; Zhu, Tang, Ma, & He, 2023) and preliminary trials, three independent variables: temperature (A1), time (A2), and S: L (A3) were used for the BBD optimization trials for AC-PT hemicellulose recovery (xylose) from CW biomass. Similarly, for EH, three independent variables, temperature (EH1), substrate loading (EH2), and time (EH3) were selected for enzymatic hydrolysis (XOS) from AC-PT extracted xylan with Design Expert-13 software (v13.05.0), developed by State-Ease, Minnesota, USA. The AC-PT and EH independent variable range is reported in **Table S1**.

**Table S1.** Experimental parameters for AC-PT treatment of CW and enzymatic hydrolysis of xylose (EH), their values at various levels

| Autoclave hydrothermal pretreatment | | | | Enzymatic hydrolysis | | | |
| --- | --- | --- | --- | --- | --- | --- | --- |
| Factors  (AC-PT) | Levels | | | Factors  (EH) | Levels | | |
|  | +1 | 0 | -1 |  | +1 | 0 | -1 |
| Temperature (°C) | 100 | 150 | 200 | Temperature (°C) | 40 | 50 | 60 |
| Time (min) | 10 | 20 | 30 | Enzyme loading (U) | 15 | 25 | 35 |
| S: L ratio (w/v) | 1: 20 | 1: 40 | 1: 60 | Time (h) | 12 | 30 | 48 |

A quadratic polynomial design was utilized to fit the experimental results for the extraction procedure (Eq. 1):

$$Y=\beta o+\sum_{i=1}^{k} \beta_{i}X_{i} +\sum_{i=1}^{k} \beta_{ii}X_{ii} + \sum_{i=1,i<j}^{k-1} \sum_{i=2}^{k} B_{ij}X_{i}X_{j} ( 1)$$

The observed output for every combination of levels of factors is represented by Y. The β_i, β_ii, and β_ij indicate the coefficients of linear, interaction, and quadratic parameters, respectively, and βo denotes the intercept. The independent variable is denoted by X, and the number of variables by k. The linear terms are labelled as A, B, and C, interaction terms as AB, AC, and BC, whereas the quadratic terms are A^2^, B^2^, and C^2^. The RSM was primarily employed to accurately represent the role of independent operational parameters on the response (reducing sugar and XOS yield). All independent variables were set to three distinct levels, namely +1, 0, and -1. This facilitates the assessment of interaction effects among diverse factors using the BBD methodology.

*S2.2 MALDI–TOF MS* *analysis*

XOS were characterized using MALDI–TOF mass spectrometry (Synapt XS HD, Waters, UK). Samples were mixed with 2,5-dihydroxybenzoic acid (DHB) matrix and analyzed in positive-ion reflectron mode. The mass spectra (MS) were obtained in the range of m/z 100 to 2000 Da, and the ions were identified as sodium adducts ([M+ Na+]) using a mass spectrometer. The oligomers were identified based on their mass and included an increment of 132 Da per xylose residue. The spectra were processed using Waters MassLynx software (Waters, UK).

*S2.3 Prebiotic Digestibility: Simulation*

A method previously described by (Minekus et al., 2014) was used to perform *in vitro* digestion of the XOS. In the oral phase of the digestion process, the XOS were mixed with 2 mL SSF (simulated saliva fluid) electrolyte solution (15.1 mmol/L KCl, 3.7 mmol/L KH_2_PO_4_, 13.6 mmol/L NaHCO_3_, 0.15 mmol/L MgCl_2_(H_2_O) 6, 0.06 mmol/L (NH_4_)2CO_3_, 1.5 mmol/L CaCl_2_(H_2_O)_2_, pH 7.0) was mixed with 80 mg XOS, an oral enzyme (α-amylase from human saliva Type IX-A, 75 U/mL) and 0.75 mM CaCl_2_. The reaction occurred at 37 °C for 2 minutes and was then terminated by boiling for 5 min. In the gastric phase of the digestion process, the oligosaccharides were mixed with 2 ml SGF (simulated gastric fluid) electrolyte solution (6.9 mmol/L KCl, 0.9 mmol/L KH_2_PO_4_, 25 mmol/L NaHCO_3_, 47.2 mmol/L NaCl, 0.1 mmol/L MgCl_2_(H_2_O)_6_, 0.5 mmol/L (NH_4_)2CO_3_, 0.15 mmol/L CaCl_2_(H_2_O)2, pH 7.0) was mixed with 80 mg XOS, 2000 U/mL of gastric enzymes (pepsin from porcine gastric mucosa type 3200-4500 U/mg protein), and 0.075 mM CaCl_2_.

The reaction occurred at 37 °C for 2 hours and was then terminated by boiling for 5 min. In the intestinal phase of the digestion process, the oligosaccharides were mixed with 2 ml SIF (simulated intestinal fluid) electrolyte solution (6.8 mmol/L KCl, 0.8 mmol/L KH_2_PO_4_, 85 mmol/L NaHCO_3_, 38.4 mmol/L NaCl, 0.33 mmol/L MgCl_2_(H_2_O)_6_, 0.6 mmol/L CaCl2(H_2_O)_2_, pH 7.0) was mixed with 80 mg XOS, and 100 U/mL of pancreatin (from porcine pancreas). The reaction occurred at 37 °C for 4 hours and was then terminated by boiling for 5 min. The next step of digestion took place at the intestinal brush border membrane (BBM). The BBM was isolated from the small intestines of pigs. BBM vesicles were mixed with 2 mL fresh SIF, 80 mg of XOS, and 2.89 U/mL (maltase activity) BBMV enzyme. The reaction occurred at 37 °C for 4 h and was then terminated by boiling for 5 min. The digestion ratios of the oligosaccharides from the oral, gastric, intestinal and BBMV phases were determined by measuring the free sugar released during digestion. The residual XOS concentration was determined by HPLC.

*S2.4 Energy Consumption*

The use of steam as an extraction tool that utilises electrical energy requires calculations to determine the amount of energy consumed. The electric consumption of the AC-PT was calculated on the basis of power consumption and extraction time. The general equation used to determine the electrical energy consumption is as follows:

$E_{C}=\frac{P X t}{36000}$	(2)

Where Ec is the electric consumption (kWh), P is the power consumption (W), and t is the extraction time (s).

Additionally, the relative electric consumption from the AC-PT method can be calculated using Eq. (3).

$E_{C}*=\frac{E_{c}}{m}$	(3)

Where EC* is the relative consumption (kWh g^−1^), and m is the mass of the resulting xylose (g).

*S2.5 CO_2_ emission*

The CO_2_ emission during the process is necessary to show the impact on the environment. The emission of CO_2_ is calculated based on 1 kWh of coal or fossil fuel energy, which releases 800 g of CO_2_ into the atmosphere during combustion. This CO_2_ emission can be expressed by Eq. (4)

$E_{{CO}_{2}}= \frac{E_{C} X 800}{1000}$	(4)

Where ECO_2_ is the CO_2_ emission (kg), Ec is the electric consumption (kWh), P is the power consumption (W), and t is the extraction time (s). In addition, the relative CO_2_ emission from the AC-PT method can be calculated using Eq. (4).

${E^{*}}_{{CO}_{2}}= \frac{E_{{CO}_{2}}}{m}$	(5)

Where E∗ CO_2_ is the relative CO_2_ emission (kg g−1), and m is the mass of the resulting xylose (g).

*S2.6 Plant toxicity*

An experimental germination experiment was undertaken at room temperature (25 °C) to evaluate the aquatic safety of an extracted XOS on mung beans. In a typical process, the mung beans were immersed overnight in distilled water, and five uniformly sized beans were kept in each petri plate. Three distinct groups were formed: a control group utilizing DW and another one with an XOS stock solution. Each Petri plate was allocated 10 mL of DW, followed by 5 mL of the corresponding treatment solution (XOS in this case). To ensure uniform moisture content, 5 mL of DW was administered to each plate daily. The growth and germination of the beans were monitored on days 1, 3, 5, 7, and 9, which were then utilized to determine the XOS aquatic toxicity on plants (Rana, Kumar, Dhiman, Wang, & Lai, 2026).

*S2.7 Human cell line cytotoxicity (cell viability)*

Normal human colon epithelial cells (NCM460) were obtained from a certified cell repository, and cells were propagated in Dulbecco’s Modified Eagle’s Medium (DMEM) enriched 1 % penicillin-streptomycin and with 10 % fetal bovine serum (FBS), and continued in a humidified incubator at 37 °C with 5 % CO₂. Cells were sub-cultured at 70–80 % confluency using trypsin–EDTA. 96-well plates were seeded with NCM460 cells and allowed to adhere overnight at a density of 1 × 10⁴ cells per well. Cells were subsequently exposed to XOS at doses of 20, 50, 75, 100, and 300 µg/mL for a duration of 24 h. Untreated cells functioned as the negative control. A positive control (Triton X-100 (0.1 %) was employed to induce complete membrane disruption and represent maximal cytotoxicity. After treatment, 20 µL of MTT solution (5 mg/mL in PBS) was introduced to each well, and the plates were maintained at 37 °C for 4 h. Once the resultant formazan crystals were mixed in 150 µL of dimethyl sulfoxide (DMSO), absorbance at 570 nm was measured using a microplate reader.

*S2.8 Antimicrobial activity of XOS*

The agar well diffusion assay was used to assess the XOS's antibacterial effectiveness against microorganisms (Yahyaoui et al., 2025). The microorganisms evaluated comprised two gram-negative bacteria: *Escherichia coli* (MTCC 1302) and *Salmonella spp.* (MTCC 3217), alongside two gram-positive bacteria: *Bacillus cereus* (MTCC 8527) and *Staphylococcus aureus* (MTCC 3160). Mueller-Hinton agar was used to seed the bacteria at a concentration of 106 CFU/mL. Agar media wells were filled with the test medium (100 μL), while the negative control was 100 μL of the inoculation-free medium. A positive control against bacteria was gentamicin (1 mg/mL) from Sigma-Aldrich, MO, USA. The medium was maintained for 24 hours at 37 °C for bacterial growth. The inhibitory zones at the millimetre scale surrounding the wells were quantified.

*S2.9 Diphenyl-1-Picrylhydrazyl (DPPH) Radical Scavenging Test*

The free-radical scavenging activity of xylan and XOS was estimated by the standard DPPH assay method. 1mL of the sample was added to a mixture of 4mL of 80 % methanol and 1mL (containing 1mmol) DPPH. Furthermore, the mixture was incubated at 25 °C for 20 min. The optical density (OD) at 517 nm was recorded on a UV-Vis spectrophotometer (Shimadzu UV-1900) to determine the μmoles of DPPH scavenged by xylan and XOS extract. The following formula was used to determine the radical-scavenging activity.

*DPPH Scavenging activity* (%) = *OD_Control_* -*OD_sample_ /OD_Control_ X* 100	(6)

*S2.10 Discussion on BBD analysis of AC-PT treatment*

An ANOVA analysis of the experimental data produced the following quadratic polynomial equation:

Y_RS_=53.67 +6.79A + 0.9500B +6.25C -7.07AB + 22.90AC + 19.52BC +16.69A^2^ -5.36B^2^ -6.70C^2^	(7)

Y_TS_=68.11 + 30.06A + 1.59B + 15.98C +0.9500AB + 8.55AC -0.3400BC + 1.42A^2^ -0.2747B^2^ + 1.08C^2^	(8)

**Tables S3** and **S4** (for xylose and total sugars) show the p-value (<0.0001), which indicates that regression terms exhibited statistical significance. The model fitness and accuracy were validated by the coefficient of determination (R^2^=0.9993) and adjusted R^2^ (R^2^adj.=0.9984) for xylose and (R^2^=0.9995) and adjusted R^2^ (R^2^adj.=0.9988) for total sugar. Also, CV, i.e. coefficient of variation of 1.28 % (xylose) and 1.22 % (total sugar), represents excellent precision and consistency of experimental observations and significance of the model for AC-PT. The F-value of 1096.23 and 1503.75, with low p-values (p<0.0001) for xylose and total sugar for AC-PT, respectively, was obtained, again suggesting a statistically significant model. It was thus determined that the polynomial model was a good fit with excellent accuracy and reliability. 99.5 % degree of precision for RSM was achieved for xylose extraction by AC-PT, demonstrating a good predictive model capability and close agreement with the experimental findings under the most optimal set of parameters.

The regression equation produces the 2D-contour and 3D-response surface plots that show the interactions between factors (two variables in each) (**Figure 2** and **S1**). The maximum xylose yield (99.13 mg/g) was observed at optimized parameters of 200 °C, 20 min, and 1: 60 solid-liquid ratios. The autoclave hydrothermal pretreatment primarily attacks hemicellulose, not cellulose. The heating induces the cleavage of β-1,4-xylan linkage with the release of acetyl groups. Additionally, hydronium ions are generated autocatalytically, which can further increase depolymerization to produce soluble sugar as xylose (Hossain, Therasme, Volk, Kumar, & Kumar, 2024). As analysed from **Figure 2 (a)**, the temperature and time have a moderately strong quadratic interaction. The xylose yield increases markedly with temperature increase from 100 to 200 ^o^C and quite moderately with reaction time. The elliptical contour pattern of 2D plots also signifies a significant interaction. The prolonged pretreatment improves the solubilization of hemicellulose, but mainly at elevated temperatures owing to intensive hemicellulose hydrolysis (H. Li et al., 2015). Secondly, the solid–liquid ratio increase enhanced the xylose yield, likely because of improved mass transfer and lesser saturation limitations. The temperature remained the most dominant factor and showed moderate to strong quadratic interaction with the other two variables.

**Table S2.** BBD evaluating the effect of different AC-PT combinations on xylose (reducing sugar) and total sugar yield of CW

| **Run** | **AC-PT** | | | **Reducing Sugar Xylose (mg/g)** | | **Total Sugar**  **(mg/g)** | |
| --- | --- | --- | --- | --- | --- | --- | --- |
|  | **A1** | **A2** | **A3** | **Actual**  **Values*** | **Predicted**  **Values** | **Actual**  **Values*** | **Predicted**  **Values** |
| **1** | 200 | 20 | 1: 20 | 40.89 | 41.30 | 76.18 | 76.14 |
| **2** | 100 | 10 | 1: 40 | 60.06 | 60.91 | 38.72 | 38.55 |
| **3** | 150 | 20 | 1: 40 | 54.19 | 53.67 | 68.26 | 68.11 |
| **4** | 150 | 20 | 1: 40 | 53.02 | 53.67 | 68.85 | 68.11 |
| **5** | 150 | 30 | 1: 20 | 27.07 | 27.50 | 55.01 | 54.87 |
| **6** | 150 | 30 | 1: 60 | 78.66 | 79.05 | 85.41 | 86.15 |
| **7** | 200 | 10 | 1: 40 | 88.65 | 88.62 | 96.00 | 96.77 |
| **8** | 100 | 20 | 1: 20 | 73.97 | 73.51 | 32.21 | 33.12 |
| **9** | **200** | **20** | **1: 60** | **99.13** | **99.59** | **126.13** | **125.21** |
| **10** | 150 | 10 | 1: 60 | 38.54 | 38.10 | 83.51 | 83.64 |
| **11** | 150 | 20 | 1: 40 | 53.98 | 53.67 | 67.68 | 68.11 |
| **12** | 150 | 20 | 1: 40 | 53.07 | 53.67 | 67.97 | 68.11 |
| **13** | 200 | 30 | 1: 40 | 77.25 | 76.39 | 101.70 | 101.86 |
| **14** | 100 | 30 | 1: 40 | 76.92 | 76.94 | 40.62 | 39.84 |
| **15** | 100 | 20 | 1: 60 | 40.63 | 40.21 | 47.95 | 47.98 |
| **16** | 150 | 20 | 1: 40 | 54.09 | 53.67 | 67.80 | 68.11 |
| **17** | 150 | 10 | 1: 20 | 65.05 | 64.65 | 51.75 | 51.00 |

**A1:** Temperature (ºCelsius), **A2:** Time (minutes) **A3:** S: L Ratio *mean ± SD (n=3)

**Table S3 (a):** Results of RSM-based ANOVA highlighting the impact of AC-PT process conditions on xylose yield **(b)** Results of RSM-based ANOVA highlighting the impact of AC-PT process conditions on total sugar

| (A) **Source** | **Sum of Squares** | **df** | **Mean Square** | **F-value** | **p-value** |
| --- | --- | --- | --- | --- | --- |
| **Model** | 5973.89 | 9 | 663.77 | 1096.23 | < 0.0001^***^ |
| A-Temperature | 369.10 | 1 | 369.10 | 609.59 | < 0.0001^***^ |
| B-Time | 7.22 | 1 | 7.22 | 11.92 | 0.0106^*^ |
| C-S: L Ratio | 312.25 | 1 | 312.25 | 515.69 | < 0.0001^***^ |
| AB | 199.66 | 1 | 199.66 | 329.74 | < 0.0001^***^ |
| AC | 2096.72 | 1 | 2096.72 | 3462.80 | < 0.0001^***^ |
| BC | 1524.90 | 1 | 1524.90 | 2518.42 | < 0.0001^***^ |
| A² | 1172.52 | 1 | 1172.52 | 1936.44 | < 0.0001^***^ |
| B² | 121.08 | 1 | 121.08 | 199.97 | < 0.0001^***^ |
| C² | 189.15 | 1 | 189.15 | 312.39 | < 0.0001^***^ |
| **Residual** | 4.24 | 7 | 0.6055 |  |  |
| Lack of Fit | 2.91 | 3 | 0.9710 | 2.93 | 0.1629^ns^ |
| Pure Error | 1.33 | 4 | 0.3313 |  |  |
| **Cor Total** | 5978.13 | 16 |  |  |  |
| R^2^ | 0.9993 |  |  |  |  |
| Adjusted R^2^ | 0.9984 |  |  |  |  |
| Predicted R^2^ | 0.9919 |  |  |  |  |
| C.V | 1.28 % |  |  |  |  |

***p < 0.05:** significant**, **p < 0.01:** highly significant**, ***p < 0.001:** extremely significant, ^ns^**p > 0.05:** not-significant

| (B) **Source** | **Sum of Squares** | **df** | **Mean Square** | **F-value** | **p-value** |
| --- | --- | --- | --- | --- | --- |
| **Model** | 9605.10 | 9 | 1067.23 | 1503.75 | < 0.0001^***^ |
| A-Temperature | 7230.63 | 1 | 7230.63 | 10188.08 | < 0.0001^***^ |
| B-Time | 20.35 | 1 | 20.35 | 28.68 | 0.0011^**^ |
| C-S: L Ratio | 2043.20 | 1 | 2043.20 | 2878.91 | < 0.0001^***^ |
| AB | 3.61 | 1 | 3.61 | 5.09 | 0.0587^ns^ |
| AC | 292.58 | 1 | 292.58 | 412.25 | < 0.0001^***^ |
| BC | 0.4624 | 1 | 0.4624 | 0.6515 | 0.4461^ns^ |
| A² | 8.52 | 1 | 8.52 | 12.01 | 0.0105^*^ |
| B² | 0.3178 | 1 | 0.3178 | 0.4478 | 0.5248^ns^ |
| C² | 4.94 | 1 | 4.94 | 6.96 | 0.0336^*^ |
| **Residual** | 4.97 | 7 | 0.7097 |  |  |
| Lack of Fit | 4.10 | 3 | 1.37 | 6.27 | 0.0541^ns^ |
| Pure Error | 0.8707 | 4 | 0.2177 |  |  |
| **Cor Total** | 9610.07 | 16 |  |  |  |
| R^2^ | 0.9995 |  |  |  |  |
| Adjusted R^2^ | 0.9988 |  |  |  |  |
| Predicted R^2^ | 0.9930 |  |  |  |  |
| C.V | 1.22 % |  |  |  |  |

***p < 0.05:** significant**, **p < 0.01:** highly significant**, ***p < 0.001:** extremely significant, ^ns^**p > 0.05:** not-significant

(a)

(b)

(c)

**Fig. S1 (a)** 3D contour plots and 2D response surface plots for total sugar in CW after AC-PT **(b)** 3D contour and 2D response surface plots illustrating xylose yield from CW after AC-PT; **(c)** Diagnostic plots of predicted vs actual analysis for (a) reducing sugar (xylose), (b) total sugar for AC-PT, and (c) enzymatic hydrolysis of XOS, respectively

(a)

(b)

**Fig. S2 (a)** HPLC chromatogram of xylose (obtained by AC-PT) **(b)** HPLC chromatogram of XOS (obtained by enzymatic hydrolysis of CW)

**Figure S3:** LC–ESI–MS positive-ion spectra of XOS showing characteristic molecular ions and fragment ions corresponding to xylose oligomers of varying degrees of polymerization (X2-X6)

**Figure S4:** LC–ESI–TOF–MS positive-ion spectra of purified XOS showing dominant molecular ions at *m/z* 282.31 and 284.32, together with minor adduct and fragment ions

**Table S4. Approximate cost evaluation of sequential process based XOS production from CW waste**

| **Material/reagent** | **Unit cost (INR)** | **Amount used (kg)/No of units** | **Effective cost (INR)** |
| --- | --- | --- | --- |
| Cauliflower waste | ---- | 0.050 | ….. |
| Ultrafiltration tube (Purchase basis) | 300 | 2 | 600 |
| Nanofiltration tube (Purchase basis) not on replacement basis | 300 | 2 | 600 |
| Enzyme | 1200/1000U | 100 units | 120 |
| **Energy cost (as per kWh usage)** |  |  | **Cost** |
| Drying | – | – | 50 |
| Grinding | – | – | 20 |
| Centrifugation/purification |  |  | 30 |
| Autoclave | – | – | 40 |
| Enzymatic hydrolysis | – | – | 50 |
| Centrifugation | – | – | 25 |
| **Total** |  |  | **1535** |
|  |  |  | **Effective cost per g of XOS produced= 345 INR** |

**Table S5.** Concentration of short-chain fatty acids (mg/mL)

| **Acid** | **S1 (mg/mL)** | **S4 (mg/mL)** | **S3 (mg/mL)** | **S2** | **S5** |
| --- | --- | --- | --- | --- | --- |
| Acetic acid | 2.03 ± 0.31 | 2.25 ± 0.11 | 1.99 ± 0.23 | 1.78 ± 0.18 | 1.69 ± 0.07 |
| Propionic acid | 0.12 ± 0.00 | 0.16 ± 0.01 | 0.08 ± 0.00 | 0.05 ± 0.01 | 0.12 ± 0.00 |
| Butyric acid | 0.05 ± 0.00 | 0.04 ± 0.00 | 0.03 ± 0.01 | 0.03 ± 0.00 | 0.02 ± 0.00 |
| Iso-butyric acid | – | 0.01 ± 0.00 | – | ---- | – |
| Isovaleric acid | 0.02 ± 0.00 | 0.02 ± 0.00 | – | ----- | – |
| Total | 2.22 ± 0.31 | 2.48 ± 0.11 | 2.10 ± 0.23 | 1.86 ± 0.18 | 1.83 ± 0.07 |

Values are expressed as mean ± standard deviation (SD) of triplicate determinations (n = 3)

**Table S6:** Cauliflower waste-derived XOS digestibility percentage

| **Phase** | **Digestibility Per cent (%)** | **Percentage of XOS remaining (%)** |
| --- | --- | --- |
| Oral | 0.78 ± 0.05 | 99.2 ± 0.05 |
| Gastric | 1.47 ± 0.06 | 97.75 ± 0.06 |
| Intestinal | 2.92 ± 0.14 | 94.83 ± 0.16 |
| BBMV | 4.55 ± 0.16 | 90.28 ± 0.16 |
| Total | 9.72 ± 0.16 | 90.28 ± 0.16 |

Values are expressed as mean ± standard deviation (SD) of triplicate determinations (n = 3)
